# Obesity-induced metabolic reprogramming of the tumor immune microenvironment: mechanisms, spatial niches, and immunotherapy response

**DOI:** 10.3389/fimmu.2026.1901774

**Published:** 2026-07-22

**Authors:** Yikang Xu, Jiaxing Yang

**Affiliations:** Department of Gastrointestinal and Colorectal Surgery, General Surgery, The First Hospital of Jilin University, Changchun, Jilin, China

**Keywords:** immune checkpoint blockade (ICB), metabolic reprogramming, obesity, spatial immunometabolic niches, tumor immune microenvironment (TIME)

## Abstract

Obesity is a major global health challenge and an established risk factor for cancer. Beyond increasing tumor incidence, obesity reshapes the tumor immune microenvironment (TIME) through systemic metabolic reprogramming, adipokine dysregulation, chronic inflammation, and gut microbiota alterations. These systemic changes create spatially organized immunosuppressive metabolic niches, including adipocyte-rich, hypoxic/lactate-enriched, myeloid-dense, and CAF/ECM barrier regions. Such niches restrict effector T-cell and NK-cell function while supporting regulatory T cells, tumor-associated macrophages (TAMs), and myeloid-derived suppressor cells (MDSCs), collectively promoting tumor progression and therapy resistance. Obesity also generates context-dependent effects on immune checkpoint blockade (ICB), a phenomenon known as the “obesity paradox, ” in which immune suppression coexists with increased checkpoint dependency. Understanding how obesity modulates tumor cell metabolism, immune-cell metabolic fitness, and stromal remodeling is essential for designing effective interventions. Therapeutic strategies combining metabolic modulation, ICB, lifestyle intervention, and microbiota-targeted therapies may convert obesity-driven immune suppression into actionable vulnerabilities. Integrating systemic metabolic indicators, immune-cell signatures, and spatial biomarkers will enable precision stratification of patients and inform combination immunotherapy strategies in obesity-associated cancers.

## Highlights

- Obesity induces systemic metabolic and inflammatory reprogramming that reshapes the TIME.
- Spatial metabolic niches restrict effector immunity while supporting suppressive immune populations.
- Combined metabolic and immunotherapeutic interventions offer potential strategies to overcome obesity-associated immune suppression.

## 1 Introduction

Obesity has become a major global public health challenge and is increasingly recognized as an important modifiable risk factor for cancer development and progression (1–5). Beyond its traditional definition as excessive adiposity, obesity represents a complex systemic metabolic and inflammatory state characterized by insulin resistance, dysregulated adipokine secretion, lipid overload, chronic low-grade inflammation, altered gut microbiota, and impaired immune homeostasis (6–10). These systemic alterations not only increase cancer susceptibility but also shape tumor evolution by modifying the metabolic and immune landscape in which malignant cells grow. Accordingly, obesity should not be viewed merely as a background comorbidity in oncology, but rather as an active biological force that can remodel the tumor ecosystem.

The tumor immune microenvironment (TIME) is highly sensitive to metabolic perturbations. Immune cells require precise metabolic programs to support activation, proliferation, effector function, memory formation, and immune surveillance (11–15). In obesity, excessive nutrient availability and lipid accumulation can paradoxically create a metabolically hostile tumor microenvironment, in which tumor cells and stromal cells compete with immune cells for key nutrients while producing suppressive metabolites. Ringel et al. demonstrated that obesity reshapes tumor metabolism by increasing tumor-cell lipid uptake and fatty acid utilization, thereby limiting metabolic fitness and antitumor activity of CD8+ T cells within the tumor microenvironment (16). This finding provides a conceptual foundation for understanding obesity as a driver of metabolic competition between cancer cells and immune cells.

In addition to direct nutrient competition, obesity promotes the expansion and functional remodeling of immunosuppressive cell populations. Myeloid-derived suppressor cells (MDSCs), tumor-associated macrophages (TAMs), regulatory T cells, and metabolically exhausted T cells can all be influenced by obesity-associated inflammatory and metabolic cues (17, 18). For example, Gibson et al. showed that obesity-associated MDSCs promote apoptosis of tumor-infiltrating CD8+ T cells and contribute to immunotherapy resistance in breast cancer (19). This study highlights how obesity-induced myeloid reprogramming can suppress cytotoxic T-cell immunity and weaken antitumor therapeutic responses. Similarly, Dyck et al. reported that the obese tumor microenvironment suppresses CD8+ T-cell infiltration and effector function, further supporting the view that obesity can generate immune-excluded or immune-dysfunctional tumor niches (20).

A central paradox in this field is that obesity is generally associated with cancer-promoting inflammation, immune suppression, and worse metabolic health, yet several clinical and experimental studies have suggested that obese patients may show improved responses to immune checkpoint blockade in certain cancer types. This phenomenon, often referred to as the “obesity paradox, ” does not imply that obesity is beneficial. Rather, it reflects the context-dependent interaction between systemic metabolism, immune exhaustion, checkpoint dependency, tumor type, sex, adipose distribution, and treatment modality. Obesity may simultaneously impair baseline antitumor immunity while increasing the dependence of immune cells or myeloid populations on checkpoint pathways such as PD-1/PD-L1, thereby creating conditions in which immune checkpoint inhibitors may partially restore antitumor immune activity (21, 22).

Therefore, a comprehensive framework is needed to explain how obesity-induced metabolic reprogramming connects systemic metabolic dysfunction with local tumor immune regulation and therapeutic outcomes. In this review, we discuss obesity as a systemic metabolic state that reshapes tumor-intrinsic metabolism, adipocyte–tumor–immune crosstalk, immune-cell metabolic fitness, stromal remodeling, and spatial immunometabolic niches. We further examine how these obesity-driven alterations influence immunotherapy response, including both immune resistance and obesity-associated sensitivity to checkpoint blockade. By integrating metabolic, immunological, spatial, and translational perspectives, this review aims to provide a mechanistic framework for understanding obesity-associated tumor immune remodeling and to identify opportunities for biomarker development and metabolism-guided immunotherapeutic strategies.

## 2 Obesity as a systemic metabolic and inflammatory state

Obesity, often induced by chronic high-fat diet (HFD) consumption, represents a systemic metabolic and inflammatory condition that profoundly impacts multiple organ systems, including the tumor microenvironment. At the systemic level, obesity is characterized by hyperinsulinemia and elevated insulin-like growth factor 1 (IGF-1) signaling, which can promote cell proliferation and survival, favoring tumor initiation and progression (23–26). Excess circulating lipids, including fatty acids and cholesterol, contribute to ectopic lipid deposition in non-adipose tissues and enhance metabolic stress within the tumor immune microenvironment. Adipokine dysregulation is also a hallmark of obesity, with increased leptin levels and decreased adiponectin concentrations, which together modulate immune cell activity and inflammatory responses (27–31). Importantly, obesity is metabolically heterogeneous, and not all individuals with obesity exhibit the same degree of immune-metabolic dysfunction (32). Metabolically healthy obesity (MHO) generally refers to obesity without overt insulin resistance, dyslipidemia, hypertension, or systemic inflammatory abnormalities, whereas metabolically unhealthy obesity (MUO) is characterized by visceral adiposity, insulin resistance, adipose tissue inflammation, ectopic lipid deposition, and a more pronounced proinflammatory milieu (33, 34). This distinction is highly relevant to tumor immunity because MUO may generate stronger immunosuppressive pressure through elevated inflammatory cytokines, leptin/adiponectin imbalance, myeloid-cell activation, and impaired effector T-cell metabolic fitness. In contrast, MHO may be associated with a less severe inflammatory and immune-suppressive phenotype, although it may represent a transitional rather than permanently benign state (35, 36). Therefore, BMI alone is insufficient to capture obesity-related immune risk, and future studies should incorporate metabolic health parameters when evaluating obesity-associated TIME remodeling and immunotherapy response.

Chronic low-grade inflammation is a defining feature of obesity. Proinflammatory cytokines such as interleukin-6 (IL-6), tumor necrosis factor-alpha (TNF-α), and interleukin-1β (IL-1β) are elevated in the circulation and adipose tissue, contributing to systemic immune activation and metabolic dysregulation (37–40). This persistent inflammatory state alters immune cell differentiation, polarization, and function, creating a predisposition for immune suppression and dysregulated antitumor immunity. The gut microbiota should also be viewed as a sustained inflammatory amplifier rather than a secondary consequence of obesity (41, 42). Under obesogenic dietary conditions, gut dysbiosis, impaired intestinal barrier integrity, and altered microbial metabolite production can continuously feed systemic inflammation by promoting endotoxin exposure, myeloid-cell activation, cytokine release, and metabolic immune stress (43, 44). In this context, the gut–adipose–tumor axis forms a self-reinforcing inflammatory circuit: obesity reshapes microbial ecology, dysbiotic microbiota sustain metabolic inflammation, and these systemic signals further condition the tumor immune microenvironment toward myeloid expansion, effector T-cell dysfunction, and immune suppression.

Another layer of obesity-associated inflammation involves senescence-associated secretory phenotype (SASP) within adipose tissue (45, 46). In obese adipose depots, senescent adipocytes and senescence-like adipose tissue macrophages can release SASP-related factors, including IL-6, IL-1β, TNF-α, CCL2, CXCL8, matrix-remodeling enzymes, and other inflammatory mediators (47, 48). These signals may amplify chronic low-grade inflammation, promote monocyte and myeloid-cell recruitment, reinforce adipose tissue dysfunction, and contribute to a systemic milieu that favors tumor-promoting immune remodeling. Therefore, SASP may serve as an additional mechanistic bridge linking adipose tissue inflammation to myeloid expansion, T-cell dysfunction, stromal remodeling, and obesity-associated TIME suppression.

In addition to endocrine and inflammatory changes, obesity reshapes the composition and metabolic output of the gut microbiota, which in turn affects host immunity and tumor biology. Shao et al. demonstrated that HFD-induced alterations in gut microbial communities lead to changes in microbial metabolite production, particularly short-chain fatty acids such as butyrate, which can modulate myeloid cell differentiation and influence tumor immune surveillance (49). Chen et al. further showed that gut microbiota–derived metabolites under HFD conditions drive differentiation of polymorphonuclear myeloid-derived suppressor cells (PMN-MDSCs), promoting systemic immune suppression and accelerating tumor progression (50). Similarly, Li F et al. reported that HFD exacerbates lung cancer development through the STAT1/SLC7A11 signaling axis, highlighting the interplay between diet-induced metabolic changes and tumor cell–intrinsic pathways (51). Interestingly, metabolic and inflammatory alterations induced by obesity are not always fully reversible. Kuziel et al. demonstrated that formerly obese mice retain a “weight-loss memory” in mammary tissue, with persistent remodeling of the extracellular matrix and immune niches, suggesting that prior exposure to obesity can have lasting effects on tumor susceptibility and immune regulation even after body weight normalization (52). These observations underscore the complexity and long-lasting nature of systemic metabolic reprogramming induced by obesity, emphasizing its central role in shaping the tumor immune microenvironment.

**Figure 1:**
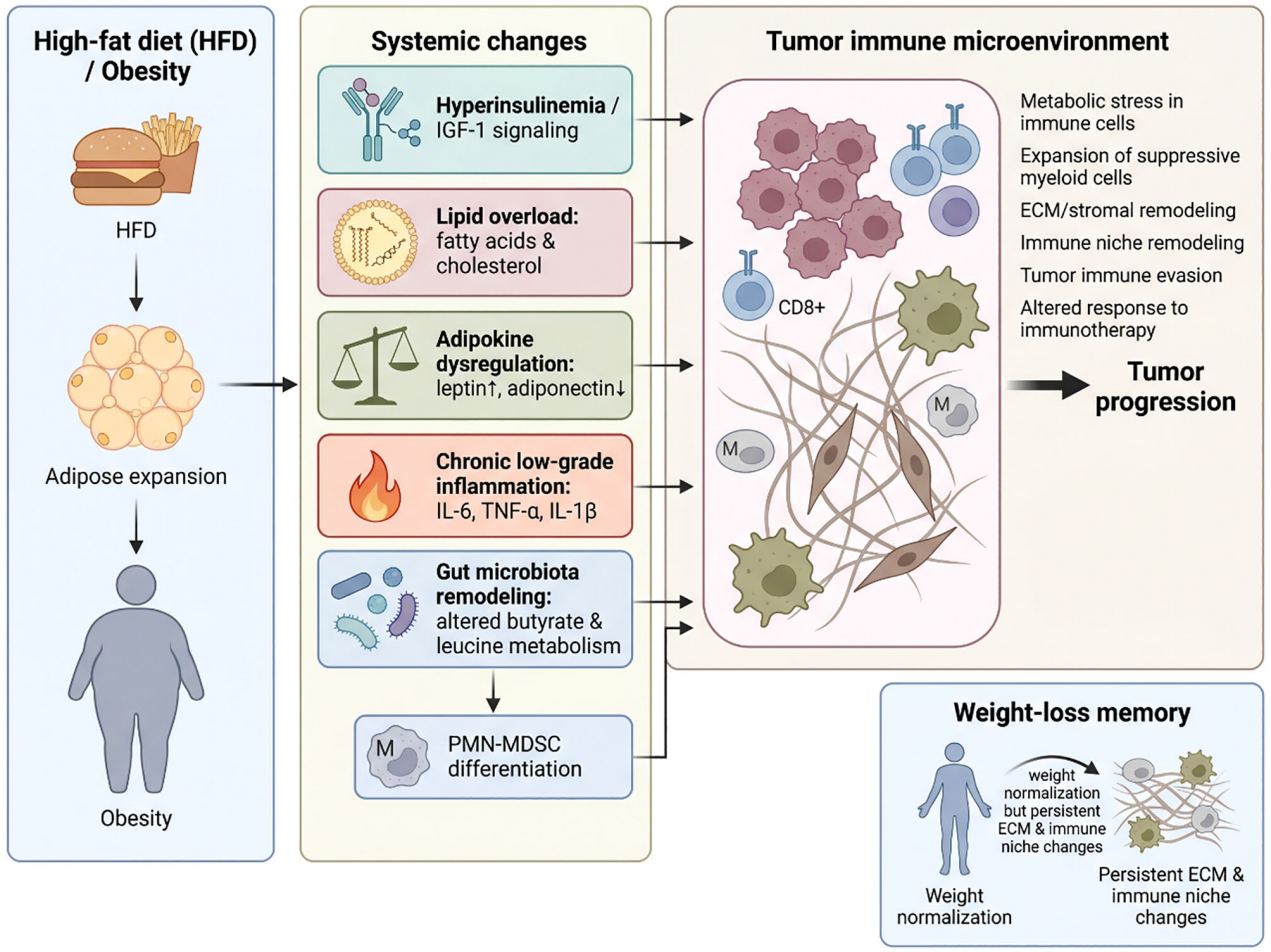
Obesity-induced systemic metabolic and inflammatory remodeling of the tumor immune microenvironment. High-fat diet/obesity drives hyperinsulinemia, lipid overload, adipokine imbalance, chronic inflammation, and gut microbiota remodeling, thereby promoting immune-cell metabolic stress, suppressive myeloid expansion, stromal remodeling, immune evasion, altered immunotherapy response, and tumor progression. Prior obesity may also leave persistent ECM and immune-niche changes after weight normalization. CD8+, cluster of differentiation 8-positive; ECM, extracellular matrix; HFD, high-fat diet; IGF-1, insulin-like growth factor 1; IL-1β, interleukin-1 beta; IL-6, interleukin-6; M, macrophage/myeloid cell; MDSC, myeloid-derived suppressor cell; PMN-MDSC, polymorphonuclear myeloid-derived suppressor cell; TNF-α, tumor necrosis factor-alpha.

Collectively, these systemic metabolic and inflammatory perturbations induced by HFD or obesity establish a predisposing environment for altered tumor immunology. They create conditions in which immune cells are metabolically challenged, myeloid suppressive populations are expanded, and tumors can exploit nutrient-rich, proinflammatory niches to evade immune surveillance. Understanding these systemic alterations is therefore critical to unraveling how obesity influences local tumor immunity and response to immunotherapy. Recent experimental studies have provided mechanistic evidence that obesity reshapes tumor metabolism, immune-cell function, stromal organization, and immunotherapy response. **Table 1** summarizes studies from 2020 onward that form the core evidence base for this review. **Figure 1** summarizes how HFD-induced obesity acts as a systemic metabolic and inflammatory driver that remodels the tumor immune microenvironment through endocrine, lipid, cytokine, and microbiota-related pathways. It also highlights the concept of “weight-loss memory, ” in which obesity-induced stromal and immune alterations may persist even after body weight normalization.

**Table 1:** Core experimental studies on obesity-induced tumor immune remodeling.

First author	Year	Cancer type/model	Key findings	PMID
Ringel AE	2020	Breast/Murine	Obesity increases tumor FA uptake → CD8+ T-cell metabolic suppression	33301708
Gibson JT	2020	Breast/Murine	Obesity expands MDSCs → CD8+ T-cell apoptosis & ICI resistance	33123173
Dyck L	2022	Melanoma/Murine	Obese TME reduces CD8+ T-cell infiltration & effector function	35103755
Lo PK	2020	Mammary gland/Murine	Single-cell atlas: obesity alters immune & stromal compartments	32785175
Yamada K	2022	Colorectal/Murine	Obesity decreases CD4+ T-cell function; Treg lipid utilization enhanced	36611881
Dudzinski SO	2021	Breast/Murine	Leptin repolarizes TAMs → modifies antitumor immunity	34772698
Bader JE	2024	Murine/Multiple	Obesity induces PD-1 on TAMs → CD8+ T-cell suppression	38867043
Pingili AK	2021	Breast/Murine	ICI reprograms systemic and TIME immunity in obese hosts	34161764
Shao X	2023	Colitis-associated cancer/Murine	HFD alters gut microbiota → butyrate → myeloid reprogramming	37781523
Li F	2023	Lung/Murine	HFD → STAT1/SLC7A11 axis → tumor progression & immune modulation	37451475
Kuziel G	2023	Mammary/Murine	Weight-loss memory: ECM & stromal remodeling persists after weight normalization	38041006
Xue C	2025	Pancreatic/Murine	Visceral adipose EVs → tumor growth & ICB resistance	41338178
Núñez-Ruiz A	2022	Breast/Murine	Adipocyte–myeloid interactions → immune suppression	35472214
Balema W	2023	Mammary/Murine	HFD modifies lymphatic niche & stromal barriers	37801190
Boufaied N	2024	Prostate/Murine	HFD + MYC → lactate-rich TME → immune suppression	38831751
Zhao G	2024	Breast/Human	Single-cell & spatial profiling of obesity-associated TIME	39300255

CD4+, cluster of differentiation 4-positive; CD8+, cluster of differentiation 8-positive; ECM, extracellular matrix; EVs, extracellular vesicles; FA, fatty acid; HFD, high-fat diet; ICB, immune checkpoint blockade; ICI, immune checkpoint inhibitor; MDSCs, myeloid-derived suppressor cells; MYC, MYC proto-oncogene; PD-1, programmed cell death protein 1; PMID, PubMed identifier; SLC7A11, solute carrier family 7 member 11; STAT1, signal transducer and activator of transcription 1; TAMs, tumor-associated macrophages; TIME, tumor immune microenvironment; TME, tumor microenvironment; Tregs, regulatory T cells.

## 3 Obesity-driven tumor-intrinsic metabolic reprogramming

Obesity not only alters systemic metabolism and immune homeostasis but also directly reshapes the metabolic programs of tumor cells. In an obese host, tumors are exposed to a nutrient-rich yet inflammatory environment characterized by increased circulating fatty acids, altered glucose and insulin signaling, adipokine imbalance, oxidative stress, and adipose-derived extracellular vesicles. These metabolic inputs can be exploited by malignant cells to support proliferation, survival, invasion, and therapy resistance. As a result, obesity-driven tumor progression is not simply a consequence of increased energy availability; rather, it reflects adaptive metabolic reprogramming that enables tumor cells to dominate local nutrient use and remodel the tumor immune microenvironment.

One major feature of obesity-associated tumor metabolism is enhanced lipid acquisition and utilization. Increased availability of circulating fatty acids and tumor-adjacent adipocyte-derived lipids can promote fatty acid uptake, lipid droplet formation, and fatty acid oxidation (FAO) in cancer cells (53–57). These adaptations provide tumor cells with bioenergetic substrates and biosynthetic intermediates while allowing them to compete more effectively with immune cells in the tumor microenvironment. Ringel et al. provided direct experimental evidence that obesity reshapes nutrient partitioning within tumors by increasing tumor-cell lipid uptake and fatty acid metabolism. This metabolic shift reduced the availability of lipids for infiltrating CD8+ T cells, impairing their metabolic fitness and antitumor function (16). This study established a key concept for the field: obesity can drive tumor-intrinsic metabolic dominance, thereby converting nutrient abundance at the systemic level into nutrient deprivation and functional suppression at the immune-cell level. Whether obesity directly drives specific somatic mutations remains insufficiently defined, and current evidence more strongly supports obesity-associated metabolic gene-expression remodeling rather than a uniform obesity-specific mutational signature (58, 59). Transcriptomic and single-cell studies suggest that obese tumor environments can induce gene programs related to fatty acid uptake, lipid handling, oxidative phosphorylation, glycolysis, hypoxia response, antioxidant defense, inflammatory signaling, and immune regulation (60–63). For example, obesity-associated tumors may show increased expression of lipid metabolic regulators such as CD36, fatty acid-binding proteins, CPT1A-related FAO programs, and stress-response pathways that enable tumor cells to adapt to nutrient-rich but inflammatory conditions (64, 65). Single-cell and spatial transcriptomic analyses of obesity-associated breast cancer further support the existence of obesity-linked cellular and transcriptomic differences within the tumor microenvironment. Therefore, obesity-driven tumor metabolic reprogramming should be understood not only as a biochemical adaptation but also as a transcriptionally encoded metabolic state that may influence immune-cell exclusion, checkpoint dependency, and therapeutic response.

In addition to lipid metabolic rewiring, obesity can enhance glycolytic activity and lactate accumulation within tumors (66–71). Glycolytic reprogramming is a common feature of malignant progression, but obesogenic conditions may amplify this process through insulin/IGF-1 signaling, inflammatory cytokines, hypoxia, and oncogene activation. Boufaied et al. showed that an obesogenic high-fat diet cooperates with MYC to promote lactate accumulation and tumor microenvironment remodeling in prostate cancer (72). This finding is particularly important because lactate is not merely a metabolic waste product. It can acidify the tumor microenvironment, suppress cytotoxic T-cell and NK-cell function, promote regulatory T-cell and macrophage polarization, and support immune-excluded niches. Therefore, obesity-driven glycolytic remodeling may provide a mechanistic bridge between tumor-intrinsic oncogenic metabolism and local immune suppression. In addition to lipid and glucose metabolism, glutamine dependency may represent another important layer of obesity-driven tumor-intrinsic metabolic adaptation (73, 74). In lipid-rich and inflammatory tumor environments, cancer cells may increase glutamine uptake and glutaminolysis to sustain tricarboxylic acid cycle anaplerosis, nucleotide and amino acid biosynthesis, and redox homeostasis (75–77). This adaptation may be particularly relevant under obesity-associated oxidative stress, because glutamine-derived glutamate supports glutathione synthesis and helps tumor cells buffer lipid peroxidation and mitochondrial stress. Enhanced glutamine consumption by tumor cells may also intensify nutrient competition within the TIME, limiting glutamine availability for activated T cells and other immune populations that require glutamine for proliferation, cytokine production, and effector function (78, 79). Therefore, obesity-associated glutamine rewiring may cooperate with lipid uptake, FAO, glycolysis, and lactate accumulation to reinforce tumor metabolic dominance and immune-cell metabolic insufficiency.

Obesity-associated mitochondrial stress may also intersect with oncometabolite-driven epigenetic regulation (80, 81). Although classical oncometabolite accumulation is often linked to specific metabolic enzyme alterations, such as IDH, SDH, or FH dysregulation, obesity-related hypoxia, lipid overload, oxidative stress, and inflammatory metabolism may amplify metabolic–epigenetic crosstalk within tumors (82, 83). Oncometabolites such as 2-hydroxyglutarate, succinate, and fumarate can interfere with α-ketoglutarate-dependent dioxygenases, including TET DNA demethylases and JmjC-domain histone demethylases, thereby altering DNA methylation, histone methylation, differentiation programs, and immune-regulatory gene expression (84, 85). In the obese TIME, such metabolite–epigenetic interactions may reinforce tumor-cell plasticity, inflammatory signaling, immune evasion, and therapy resistance, although direct causal evidence in obesity-associated tumors remains limited and requires further investigation.

Obesity-associated tumors may also undergo broader metabolic adaptations involving cholesterol metabolism, mitochondrial remodeling, oxidative stress tolerance, and redox homeostasis. Excess lipid exposure increases oxidative pressure, requiring cancer cells to reinforce antioxidant pathways and mitochondrial stress responses. These adaptations may enable tumor cells to survive under nutrient fluctuation, hypoxia, inflammatory stress, and treatment-induced damage. In this context, ferroptosis evasion may represent an important but still underexplored mechanism linking obesity, lipid metabolism, and cancer therapy resistance. Because ferroptosis is driven by iron-dependent lipid peroxidation, obesity-associated lipid remodeling could theoretically sensitize tumor cells to lipid oxidative stress; however, cancer cells may counterbalance this vulnerability by enhancing antioxidant systems, glutathione metabolism, lipid desaturation, or cystine uptake (86, 87). Thus, the relationship between obesity and ferroptosis is likely context-dependent and may influence both tumor progression and therapeutic response. Adipose tissue-derived extracellular vesicles represent another mechanism by which obesity can reprogram tumor-intrinsic metabolism and immune interactions. Obese visceral adipose tissue is metabolically active and can release extracellular vesicles carrying proteins, lipids, metabolites, and nucleic acids that influence distant tumor sites. Xue et al. demonstrated that extracellular vesicles derived from obese visceral adipose tissue promote pancreatic cancer development and resistance to immune checkpoint blockade therapy (88). This study expands the concept of obesity-driven tumor metabolic remodeling beyond local adipocyte–tumor contact and suggests that adipose-derived vesicular signals can act systemically to enhance tumor progression and immunotherapy resistance.

**Figure 2:**
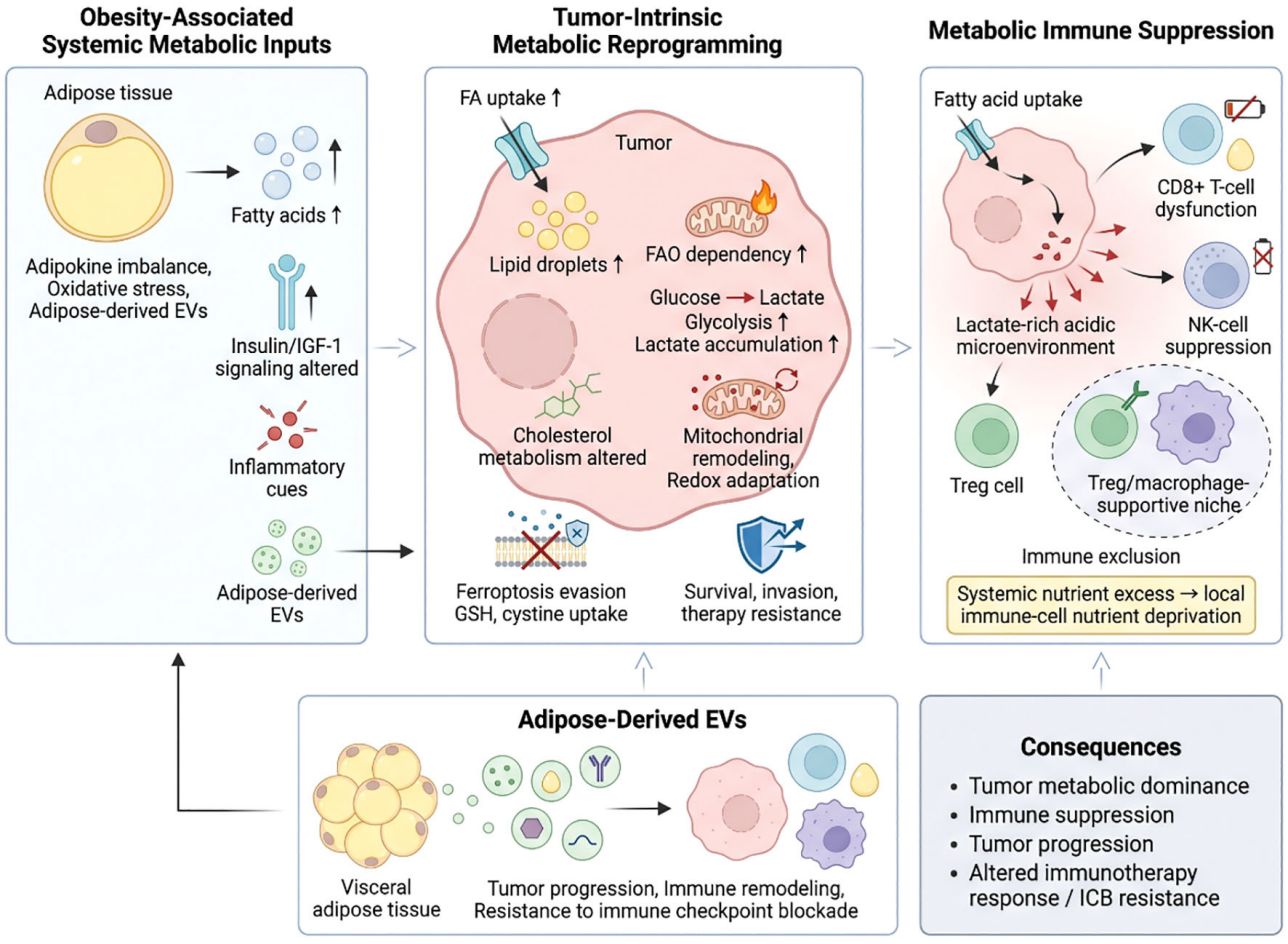
Obesity-driven tumor-intrinsic metabolic reprogramming. Obesity-associated metabolic inputs, including fatty acids, insulin/IGF-1 signaling, inflammatory cues, oxidative stress, and adipose-derived extracellular vesicles, promote tumor-cell lipid uptake, FAO dependency, glycolytic activity, lactate accumulation, mitochondrial adaptation, redox remodeling, and ferroptosis evasion. These changes enhance tumor metabolic dominance, suppress antitumor immune-cell function, and contribute to tumor progression and immunotherapy resistance. CD8+, cluster of differentiation 8-positive; EVs, extracellular vesicles; FA, fatty acid; FAO, fatty acid oxidation; GSH, glutathione; ICB, immune checkpoint blockade; IGF-1, insulin-like growth factor 1; NK cell, natural killer cell; Treg cell, regulatory T cell.

Collectively, obesity-driven tumor-intrinsic metabolic reprogramming involves coordinated changes in lipid uptake, FAO dependency, glycolytic activity, lactate accumulation, glutamine dependency, mitochondrial adaptation, redox balance, metabolic gene-expression programs, and resistance-associated pathways. These tumor-centered changes are closely linked to immune regulation because they alter nutrient availability, metabolite accumulation, stromal organization, and checkpoint dependency within the tumor microenvironment. Therefore, tumor-intrinsic metabolic adaptation should be considered a central mechanism by which obesity reshapes antitumor immunity and modifies response to immunotherapy. **Figure 2** illustrates how obesity directly rewires tumor-cell metabolism, enabling malignant cells to exploit systemic nutrient excess while creating local metabolic stress for immune cells. It highlights lipid uptake, lactate accumulation, redox adaptation, and adipose-derived extracellular vesicles as major mechanisms linking tumor-intrinsic metabolic remodeling to immune suppression and altered immunotherapy response.

## 4 Reprogramming of immune-cell metabolism in obese TIME

The antitumor immune response is tightly coupled to cellular metabolism. Effector T cells, regulatory T cells, macrophages, myeloid-derived suppressor cells, natural killer cells, and innate lymphoid cells require distinct metabolic programs to sustain activation, differentiation, cytokine production, cytotoxicity, and tissue adaptation. In obesity, excessive nutrient availability at the systemic level does not necessarily improve immune-cell function within tumors. Instead, obesity creates a metabolically distorted tumor immune microenvironment (TIME), where tumor cells, stromal cells, and suppressive myeloid populations dominate nutrient consumption and expose antitumor immune cells to lipid overload, hypoxia, lactate accumulation, oxidative stress, and chronic inflammatory signaling. These conditions drive immune-cell metabolic dysfunction and contribute to impaired tumor immune surveillance, immune exclusion, and altered sensitivity to immunotherapy. Importantly, obesity does not uniformly suppress all immune populations. Instead, immune-cell outcomes depend on the balance between activating cues and suppressive cues within specific metabolic niches (89, 90). Activating cues, such as sufficient nutrient availability, preserved mitochondrial fitness, antigen stimulation, type I interferon signaling, interleukin-12, and effective checkpoint blockade, can support cytotoxic T-cell, NK-cell, and immune-stimulatory macrophage functions (91, 92). In contrast, suppressive cues, including lipid overload, lactate accumulation, hypoxia, chronic IL-6/TNF-α/IL-1β signaling, leptin–adiponectin imbalance, TGF-β, arginine depletion, reactive oxygen species, and stromal exclusion, favor T-cell exhaustion, Treg persistence, TAM/MDSC-mediated suppression, and impaired immune surveillance. Therefore, the following sections distinguish cell-type-specific immune activation from obesity-driven immune suppression.

At the molecular level, obesity-induced immune dysfunction is coordinated by multiple nutrient- and stress-sensing pathways. mTORC1 integrates amino acid availability, insulin/IGF-1 signaling, inflammatory cytokines, and growth-factor stimulation to regulate glycolysis, protein synthesis, and effector differentiation (93, 94). Under chronic nutrient excess and inflammatory stress, persistent or dysregulated mTORC1 activity may contribute to terminal effector differentiation, impaired memory formation, and exhaustion-like T-cell states (95, 96). In contrast, AMPK and SIRT1 function as metabolic stress sensors that preserve mitochondrial fitness, fatty acid oxidation, autophagy, and redox homeostasis (97, 98). Suppression or insufficient activation of AMPK–SIRT1 signaling in lipid-rich and hypoxic niches may impair mitochondrial quality control and weaken effector immune-cell function. HIF-1α links hypoxia and inflammatory signaling to glycolysis, lactate adaptation, and immune-cell polarization, whereas PPARγ and SREBP regulate lipid uptake, lipid storage, fatty acid handling, and cholesterol metabolism. Together, these pathways translate obesity-associated nutrient excess, lipid overload, hypoxia, and inflammation into immune-cell metabolic dysfunction (99, 100).

### 4.1 CD8+ T cells

CD8+ cytotoxic T cells are among the most metabolically demanding immune populations in the TIME (101–103). Effective antitumor activity requires sufficient glucose uptake, mitochondrial fitness, amino acid availability, and lipid metabolic balance. Obesity disrupts these requirements through multiple mechanisms. Tumor cells exposed to an obese metabolic environment can increase fatty acid uptake and lipid utilization, thereby limiting nutrient availability for infiltrating CD8+ T cells. Ringel et al. showed that obesity reshapes tumor metabolism by enhancing tumor-cell fatty acid uptake and oxidation, which compromises CD8+ T-cell metabolic fitness and antitumor function (16). This study revealed that systemic nutrient excess can paradoxically translate into local immune-cell nutrient deprivation because tumor cells outcompete CD8+ T cells for metabolic resources.

In addition to nutrient competition, the obese TIME can directly suppress CD8+ T-cell infiltration and effector function. Dyck et al. reported that the obese tumor microenvironment reduces CD8+ T-cell infiltration and weakens effector activity, supporting the concept that obesity promotes immune-excluded and immune-dysfunctional tumor niches (20). Mechanistically, CD8+ T cells in obese tumors may experience restricted glycolysis, mitochondrial stress, lipid toxicity, and exposure to suppressive metabolites such as lactate. Lactate accumulation and extracellular acidification can impair T-cell receptor signaling, cytokine secretion, and cytotoxic granule release, while lipid overload may induce mitochondrial dysfunction and exhaustion-like phenotypes. Several signaling pathways may connect obesity-associated metabolic stress to CD8+ T-cell exhaustion. Chronic inflammatory cytokines, insulin/IGF-1 signaling, amino acid imbalance, and persistent antigen stimulation can dysregulate the PI3K–AKT–mTORC1 axis (104, 105). Although mTORC1 activation is required for early T-cell activation and glycolytic reprogramming, sustained mTORC1 signaling under nutrient-stressed conditions may limit memory differentiation, increase metabolic demand, and promote exhaustion-associated programs (106, 107). Hypoxia and lactate accumulation further activate HIF-1α-dependent glycolytic adaptation, but prolonged HIF-1α signaling in acidic and nutrient-restricted niches may reinforce effector dysfunction rather than productive cytotoxicity (108, 109). In parallel, AMPK and SIRT1 normally help maintain mitochondrial homeostasis, autophagy, and oxidative stress control; impaired AMPK–SIRT1 activity in the obese TIME may therefore reduce mitochondrial resilience and compromise CD8+ T-cell persistence.

Lipid overload may induce CD8+ T-cell mitochondrial dysfunction through several interconnected mechanisms (110, 111). Excess uptake of fatty acids and cholesterol can increase lipid droplet accumulation, acylcarnitine buildup, ceramide and diacylglycerol production, and mitochondrial reactive oxygen species generation (112, 113). These changes may damage mitochondrial membranes, impair electron transport chain activity, reduce mitochondrial spare respiratory capacity, and disrupt mitophagy. In addition, SREBP-driven cholesterol and lipid biosynthetic programs may alter membrane composition and T-cell receptor signaling, whereas PPAR-family lipid-sensing pathways may reshape fatty acid oxidation and differentiation states (114, 115). When lipid handling exceeds mitochondrial oxidative capacity, CD8+ T cells develop lipid toxicity, redox imbalance, reduced IFN-γ and granzyme production, and increased expression of inhibitory receptors such as PD-1, TIM-3, and LAG-3. Thus, obesity-associated lipid overload promotes T-cell exhaustion not simply by providing excess substrate, but by overwhelming mitochondrial quality control and metabolic flexibility.

These findings also provide a mechanistic basis for the context-dependent effects of obesity on immune checkpoint inhibitor (ICI) response. Thus, obesity rewires CD8+ T-cell metabolism in a manner that links nutrient competition, mitochondrial stress, lipid toxicity, and checkpoint dependency. Thus, CD8+ T-cell fate in obese tumors depends on the balance between activating signals and metabolic suppression. Antigen recognition, adequate glucose and amino acid availability, mitochondrial integrity, IL-12/type I interferon signaling, and checkpoint blockade may support cytotoxicity, whereas lipid overload, lactate-rich acidic pH, hypoxia, nutrient deprivation, mitochondrial stress, and persistent PD-1 signaling drive exhaustion and loss of effector function.

### 4.2 CD4+ T cells and regulatory T cells

Obesity also alters the balance between helper CD4+ T-cell immunity and Treg-mediated immunosuppression, although this axis has been less extensively studied than CD8+ T-cell dysfunction (116, 117). Obesity can reduce CD4+ T-cell abundance and impair helper T-cell function, thereby weakening immune coordination within the tumor microenvironment. Yamada et al. demonstrated that obesity reduces the number and immune function of CD4+ T cells, accelerating colorectal cancer progression (118, 119). This study highlights that obesity-associated immune dysfunction is not limited to CD8+ T cells or myeloid populations but also involves impaired CD4+ T-cell-mediated immune surveillance.

Regulatory T cells (Tregs) may be differentially affected by the obese TIME. Unlike effector T cells, which often rely on glycolysis during activation, Tregs can adapt to lipid-rich and lactate-rich environments by using fatty acid oxidation and oxidative phosphorylation. In obese tumors, increased fatty acid availability, chronic inflammation, and suppressive cytokine signaling may support Treg persistence and function. This metabolic flexibility allows Tregs to maintain immunosuppressive activity under conditions that impair effector T-cell function. As a result, obesity may shift the CD4+ T-cell compartment away from coordinated antitumor immunity and toward immune regulation, contributing to an increased Treg/effector T-cell imbalance. Therefore, obesity may shift the CD4+ compartment according to local metabolic and cytokine cues: IL-12- and interferon-associated signals may support helper T-cell-mediated antitumor coordination, whereas lipid-rich, lactate-rich, TGF-β-enriched, and IL-6-dominated niches favor Treg persistence and suppressive immune regulation.

### 4.3 Tumor-associated macrophages

Tumor-associated macrophages (TAMs) are central mediators of obesity-associated immune remodeling (120–126). In obese tissues, macrophages are exposed to adipocyte-derived lipids, leptin, inflammatory cytokines, hypoxia, and cell-death signals, all of which can alter their polarization and metabolic programs. Within tumors, obesity may promote M2-like, lipid-associated, or metabolically suppressive macrophage states that support tumor growth, angiogenesis, extracellular matrix remodeling, and T-cell dysfunction.

Leptin is one important adipokine connecting obesity to macrophage biology. Dudzinski et al. showed that leptin can augment antitumor immunity in obesity by repolarizing tumor-associated macrophages (127). This finding indicates that the relationship between obesity and TAM function is not uniformly suppressive; rather, adipokine signaling may produce distinct macrophage states depending on tumor context, immune composition, and therapeutic intervention. Such complexity is important when interpreting obesity-associated immune phenotypes, because the same systemic condition may generate divergent macrophage programs across cancer types. A more recent study further expanded the importance of macrophage checkpoint regulation in obesity. Bader et al. demonstrated that obesity induces PD-1 expression on macrophages, thereby suppressing antitumor immunity (95). PD-1-expressing TAMs can impair phagocytic and immune-stimulatory functions and contribute to CD8+ T-cell suppression. This finding provides a direct mechanistic explanation for how obesity may create checkpoint-dependent myeloid immunosuppression. It also helps reconcile the obesity paradox in ICI therapy: obese tumors may be immunosuppressed at baseline but more dependent on PD-1-mediated inhibitory circuits, making them potentially responsive to checkpoint blockade in selected settings.

Immune checkpoint blockade itself can remodel the obese immune landscape. Pingili et al. reported that PD-1 blockade reprograms both systemic immunity and the tumor microenvironment in obesity-associated breast cancer (128). This study suggests that therapeutic response in obese hosts may depend not only on reinvigoration of T cells but also on broader reconfiguration of myeloid, stromal, and systemic immune compartments. These findings indicate that obesity can generate metabolically distinct TAM states, including leptin-responsive and PD-1-dependent macrophage programs.

### 4.4 Myeloid-derived suppressor cells

Myeloid-derived suppressor cells (MDSCs) are another major immunosuppressive population expanded under obese conditions (129–131). Obesity-associated inflammation, altered lipid metabolism, and microbiota-derived metabolites can drive emergency myelopoiesis and promote the accumulation of granulocytic or monocytic MDSCs. These cells suppress antitumor immunity through arginine depletion, reactive oxygen species production, nitric oxide signaling, immunosuppressive cytokine release, and inhibition of T-cell proliferation and cytotoxicity.

Gibson et al. demonstrated that obesity-associated MDSCs promote apoptosis of tumor-infiltrating CD8+ T cells and contribute to immunotherapy resistance in breast cancer (19). This study places MDSCs at the center of obesity-driven immune escape, showing that obese tumors may become resistant to immunotherapy through enhanced myeloid suppression and direct elimination of cytotoxic T cells. From a metabolic perspective, MDSCs in obesity may exploit fatty acid uptake and fatty acid oxidation to sustain suppressive function within lipid-rich tumors.

Diet-induced alterations in gut microbiota can further reinforce MDSC expansion. Chen et al. showed that a high-fat diet promotes cancer progression by inducing gut microbiota-mediated leucine production and PMN-MDSC differentiation (50). These findings identify microbiota-driven amino acid metabolism as one route by which obesity reinforces PMN-MDSC differentiation and myeloid suppression.

### 4.5 NK cells and innate lymphoid cells

Natural killer (NK) cells and innate lymphoid cells (ILCs) are increasingly recognized as important but underexplored components of obesity-associated tumor immunity. NK cells provide rapid antitumor cytotoxicity and cytokine production without prior antigen priming. However, obesity can impair NK-cell metabolism, reduce cytotoxic granule release, and weaken interferon-γ production (132). Lipid accumulation, mitochondrial dysfunction, and chronic inflammatory exposure may collectively suppress NK-cell-mediated immune surveillance in obese hosts.

ILCs, particularly ILC2s, may also participate in obesity-associated immune remodeling. ILC2s are involved in type 2 immunity, tissue repair, adipose tissue homeostasis, and stromal regulation. In the tumor context, altered ILC2 function may influence eosinophil recruitment, macrophage polarization, fibrosis, and immune exclusion. Although direct mechanistic studies linking obesity, ILC2 metabolism, and cancer immunotherapy remain limited, this area represents an important emerging direction for future research. Future single-cell and spatial studies are needed to define how obesity affects rare innate lymphoid populations within tumor-specific metabolic niches.

**Figure 3:**
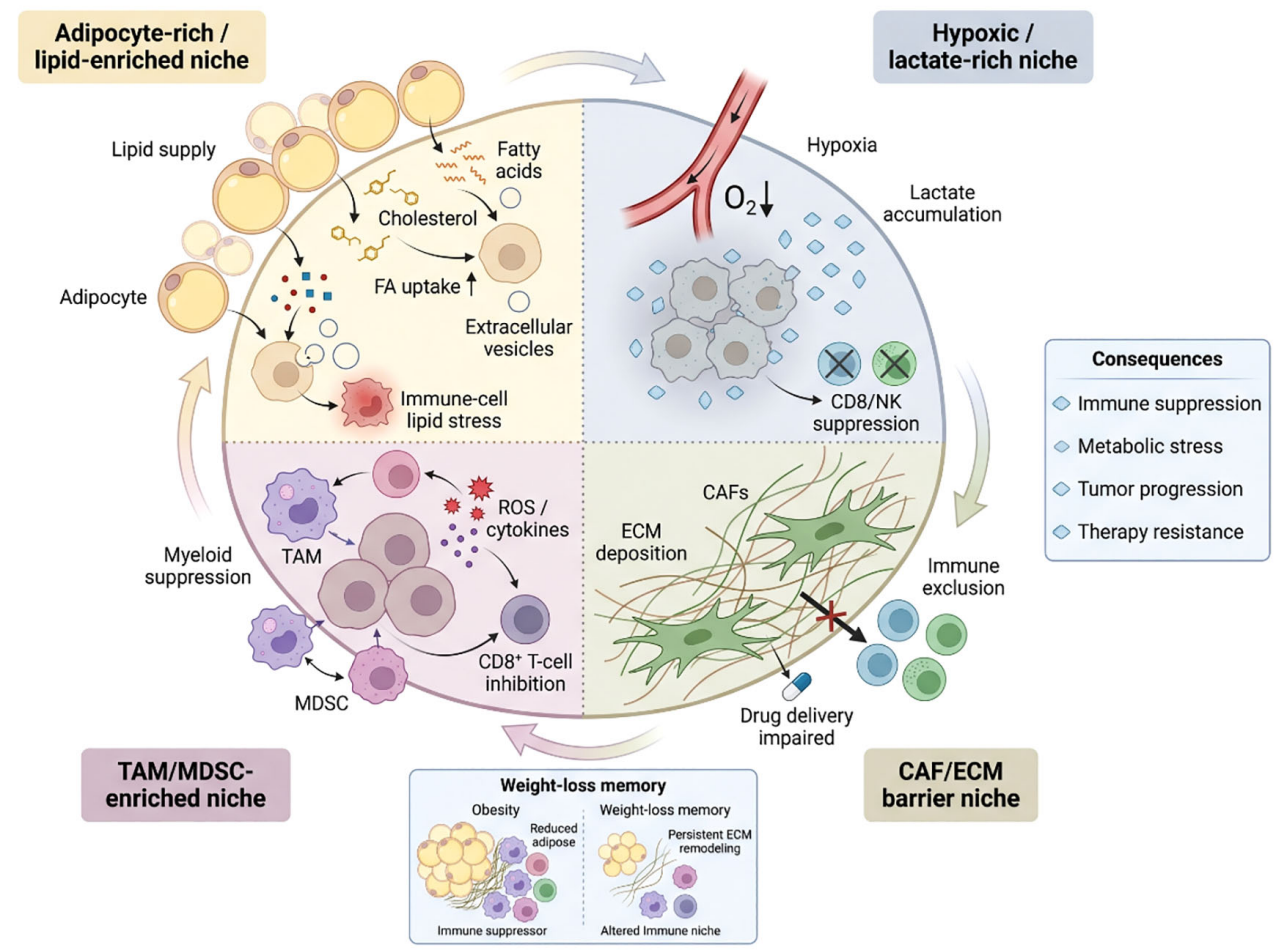
Obesity-induced immune-cell metabolic reprogramming in the TIME. Obesity creates lipid-rich, hypoxic, lactate-enriched, and inflammatory tumor niches that impair CD8+ T-cell, CD4+ T-cell, and NK-cell function while supporting Treg activity, TAM suppressive polarization, and MDSC expansion. These changes generate a myeloid-enriched immunosuppressive ecosystem that promotes immune evasion, tumor progression, and context-dependent immunotherapy response. CAFs, cancer-associated fibroblasts; CD8+, cluster of differentiation 8-positive; ECM, extracellular matrix; FA, fatty acid; MDSC, myeloid-derived suppressor cell; NK cell, natural killer cell; O_2_, oxygen; ROS, reactive oxygen species; TAM, tumor-associated macrophage.

Overall, obesity reshapes immune-cell metabolism through divergent effects on lymphoid and myeloid compartments: cytotoxic CD8+ T cells and NK cells lose metabolic fitness, Tregs adapt to lipid-rich niches, and TAMs/MDSCs acquire suppressive metabolic programs. **Table 2** summarizes these cell-type-specific alterations, and **Figure 3** illustrates how obesity shifts the TIME from effective antitumor immunity toward metabolic dysfunction and myeloid-enriched immune suppression. For NK cells, activating conditions include preserved mitochondrial function, IL-12/IL-15/IL-18 signaling, adequate nutrient supply, and effective tumor-recognition receptor engagement. In contrast, lipid accumulation, hypoxia, lactate exposure, oxidative stress, and chronic inflammatory exhaustion reduce cytotoxic granule release and interferon-γ production, shifting NK cells from rapid antitumor effectors toward metabolically impaired bystanders.

**Table 2:** Obesity-driven mechanisms in tumor cells and immune cells.

Compartment	Obesity-induced changes	Key mechanisms/metabolic pathways	References (PMID)
Tumor cells	↑ Glycolysis, ↑ Lactate	Lactate accumulation → T-cell suppression	Boufaied 2024 (38831751)
Tumor cells	↑ FA uptake, lipid droplets, FAO	Nutrient competition with CD8+ T cells	Ringel 2020 (33301708)
Tumor cells	↑ Cholesterol metabolism, oxidative stress adaptation	Ferroptosis evasion, mitochondrial remodeling	Xue 2025 (41338178)
CD8+ T cells	Exhaustion, glycolysis restriction, lipid toxicity	↓ Cytotoxicity & IFNγ, checkpoint dependency	Dyck 2022 (35103755); Ringel 2020 (33301708)
CD4+ T cells	Reduced abundance & function	Impaired helper function	Yamada 2022 (36611881)
Tregs	↑ Fatty acid oxidation	Enhanced immunosuppressive activity	Yamada 2022 (36611881)
TAMs	M2-like, lipid-associated, PD-1-dependent	Immunosuppressive, checkpoint-mediated suppression	Bader 2024 (38867043); Dudzinski 2021 (34772698)
MDSCs	Expansion, FAO ↑	Immune suppression via arginine depletion & ROS	Gibson 2020 (33123173); Chen 2024 (38709933)
NK & ILCs	Cytotoxicity ↓	Impaired metabolism and effector function	Lo 2020 (32785175)

CD4+, cluster of differentiation 4-positive; CD8+, cluster of differentiation 8-positive; FA, fatty acid; FAO, fatty acid oxidation; IFN-γ, interferon-gamma; ILCs, innate lymphoid cells; MDSCs, myeloid-derived suppressor cells; NK cells, natural killer cells; PD-1, programmed cell death protein 1; PMID, PubMed identifier; ROS, reactive oxygen species; TAMs, tumor-associated macrophages; TME, tumor microenvironment; Tregs, regulatory T cells.

↑ indicates an increase, enhancement, or upregulation, whereas ↓ indicates a decrease, impairment, or downregulation under obesity-associated conditions compared with non-obese or lean controls.

## 5 Adipocyte–tumor–immune crosstalk

Adipocytes are metabolically active cells that constitute a critical component of the tumor immune microenvironment (TIME) in obesity (93, 134–137). Beyond serving as energy storage, adipocytes actively communicate with tumor and immune cells by transferring lipids, secreting adipokines, releasing extracellular vesicles, and remodeling the extracellular matrix (ECM). These interactions collectively shape tumor progression, immune suppression, and response to therapy, highlighting adipocytes as central metabolic regulators within the obese TIME.

One primary mechanism by which adipocytes influence tumor and immune cells is through lipid transfer. Obese adipose tissue releases free fatty acids that can be taken up by neighboring tumor cells, fueling fatty acid oxidation, membrane biosynthesis, and bioenergetic needs (138–140). Simultaneously, lipid transfer can create local metabolite competition, exacerbating metabolic stress in infiltrating T cells and natural killer (NK) cells. Núñez-Ruiz et al. demonstrated that obesity-associated mammary adipose tissue drives tumor progression through adipocyte–myeloid cell interactions, where lipid-rich niches support myeloid immunosuppressive populations and limit effector T-cell activity (141). This study underscores the dual role of adipocytes in supplying nutrients for tumor growth while shaping immune-cell function. Adipokines, such as leptin and adiponectin, provide additional layers of adipocyte-mediated signaling. Leptin, typically elevated in obesity, promotes inflammatory myeloid polarization and can indirectly support tumor growth by reinforcing immune suppression. Conversely, adiponectin, which decreases in obesity, normally exerts anti-inflammatory and metabolic regulatory effects. The imbalance of these adipokines in obese hosts thus contributes to a tumor-permissive microenvironment and can modulate immune checkpoint dependency.

Extracellular vesicles (EVs) released from obese adipose tissue represent another conduit of adipocyte–tumor–immune communication (142–147). These vesicles carry lipids, proteins, and nucleic acids that can alter tumor cell metabolism and immune cell phenotype. Xue et al. reported that EVs derived from obese visceral adipose tissue promote pancreatic cancer progression and confer resistance to immune checkpoint blockade therapy (88). This work demonstrates that adipocyte-derived EVs can act systemically, transmitting obesogenic metabolic signals to distant tumors and modulating antitumor immunity. Adipocytes also influence the stromal architecture of the TIME. ECM remodeling and activation of cancer-associated fibroblasts (CAFs) are critical components of this crosstalk. Adipocyte-secreted factors can stimulate CAF proliferation and deposition of extracellular matrix proteins, creating dense stromal barriers that impede immune cell infiltration and further support immune exclusion. Balema et al. highlighted the role of obesity in modulating lymphatic vessel function and CAF-associated stromal remodeling in mammary tumors, showing that high-fat diet-induced changes in the stromal microenvironment enhance tumor growth and immune evasion (148). These findings underscore how adipocyte-mediated ECM and stromal remodeling synergize with metabolic and immunological pathways to reinforce a suppressive TIME.

**Figure 4:**
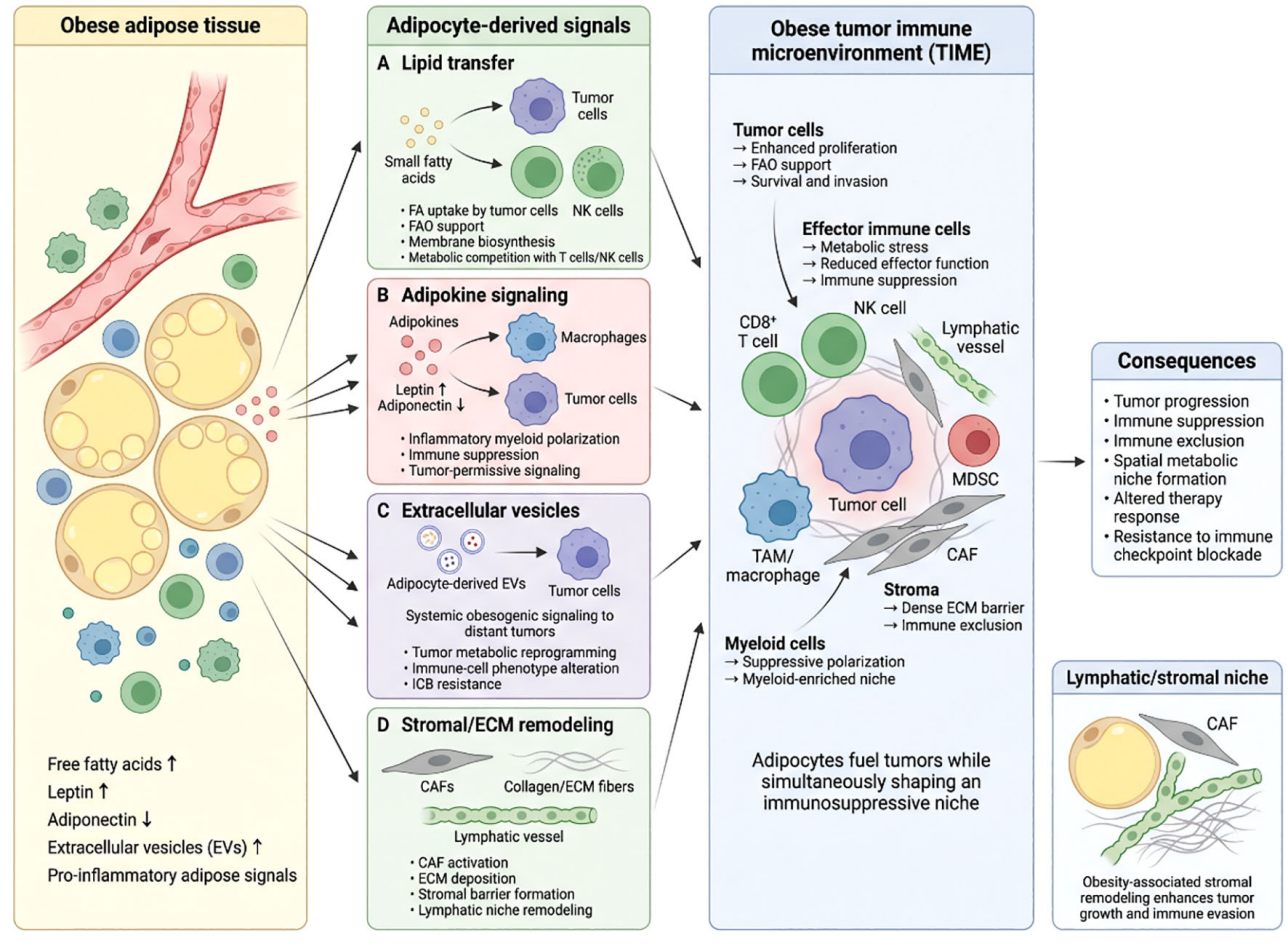
Adipocyte–tumor–immune crosstalk in the obese TIME. In obesity, adipocytes act as metabolic and signaling hubs by transferring lipids, secreting adipokines, releasing extracellular vesicles, and promoting ECM/CAF remodeling. These processes support tumor growth, suppress effector immune-cell function, reinforce stromal barriers and immune exclusion, and contribute to altered therapeutic response, including resistance to immune checkpoint blockade. CAF, cancer-associated fibroblast; CD8+, cluster of differentiation 8-positive; ECM, extracellular matrix; EVs, extracellular vesicles; FA, fatty acid; FAO, fatty acid oxidation; ICB, immune checkpoint blockade; MDSC, myeloid-derived suppressor cell; NK cell, natural killer cell; TAM, tumor-associated macrophage; TIME, tumor immune microenvironment.

In summary, adipocytes function as central metabolic and signaling hubs within the obese TIME. Through coordinated lipid transfer, adipokine secretion, EV-mediated metabolic signaling, and ECM remodeling, adipocytes promote tumor cell proliferation, shape immune suppression, and create spatially organized metabolic niches that impact therapy response. These multifaceted interactions highlight adipocytes as critical targets for interventions aimed at normalizing the tumor metabolic landscape and improving immunotherapy efficacy in obese patients. **Figure 4** summarizes how adipocytes actively shape the obese tumor immune microenvironment through metabolic, signaling, and stromal mechanisms. It highlights lipid transfer, adipokine imbalance, extracellular vesicle-mediated communication, and ECM remodeling as major pathways linking obesity to tumor progression, immune suppression, and therapy resistance.

## 6 Spatial metabolic niches in obesity-associated tumors

Obesity-associated tumor immune remodeling is not uniformly distributed across the tumor microenvironment. Instead, it occurs within spatially organized metabolic niches shaped by adipocytes, tumor cells, stromal cells, vascular structures, extracellular matrix components, and immune-cell populations. These niches are characterized by distinct nutrient availability, oxygen tension, metabolite accumulation, lipid exposure, and immune composition (149–152). Therefore, understanding obesity-associated tumor immunity requires moving beyond bulk descriptions of immune-cell abundance toward spatially resolved models of metabolic and immunological organization.

One prominent niche in obesity-associated tumors is the adipocyte-rich or lipid-enriched region. In tumors located near adipose tissue, such as breast, ovarian, pancreatic, colorectal, and prostate cancers, cancer cells can directly interact with adjacent adipocytes. These adipocyte-rich areas provide free fatty acids, cholesterol, adipokines, and extracellular vesicles that support tumor-cell metabolic adaptation (153–155). Tumor cells in these regions may increase lipid uptake, lipid droplet formation, and fatty acid oxidation, whereas nearby immune cells may experience lipid overload, mitochondrial stress, or impaired effector function. Single-cell RNA sequencing studies have begun to clarify how obesity reshapes tissue-level cellular ecosystems. Lo et al. showed that obesity induces broad transcriptomic alterations in murine mammary glands, affecting epithelial, stromal, and immune compartments (133). Although this study focused on mammary tissue rather than established tumors alone, it provides important evidence that obesity reorganizes the cellular architecture of tumor-prone tissues before or during malignant transformation.

A second spatial niche is the hypoxic and lactate-enriched region. Obesity can worsen tissue hypoxia through adipose expansion, vascular dysfunction, increased interstitial pressure, and altered perfusion. Within tumors, these conditions may intensify glycolytic metabolism and lactate accumulation. Lactate-rich regions are particularly relevant to immune suppression because extracellular acidification can inhibit CD8+ T-cell and NK-cell cytotoxicity, promote regulatory T-cell persistence, and support suppressive macrophage polarization. In obesity-associated tumors, hypoxic and lactate-rich regions may therefore function as immune-restrictive metabolic zones where tumor cells maintain growth advantages while antitumor immune cells lose metabolic fitness.

A third niche consists of TAM- and MDSC-enriched immunosuppressive regions. Obesity-associated inflammation and lipid dysregulation can promote myeloid-cell recruitment, differentiation, and suppressive activation. These myeloid-rich zones often overlap with lipid-enriched, hypoxic, or stromal-dense areas, creating local microenvironments in which CD8+ T-cell infiltration and function are constrained (156, 157). In such regions, tumor-associated macrophages and myeloid-derived suppressor cells may consume nutrients, produce reactive oxygen species, express inhibitory ligands, and release immunosuppressive cytokines. These spatially concentrated myeloid populations help explain why obese tumors may show reduced immune surveillance despite systemic inflammation.

A fourth niche involves CAF- and extracellular matrix-rich stromal barriers. Obesity can promote fibroblast activation, collagen deposition, tissue stiffness, and extracellular matrix remodeling (158, 159). These stromal barriers can physically restrict immune-cell entry, compress blood vessels, impair drug delivery, and reinforce hypoxia. Importantly, such obesity-induced stromal remodeling may persist even after weight normalization. Kuziel et al. demonstrated that formerly obese mice retain features of mammary gland and tumor microenvironment remodeling, including persistent extracellular matrix and stromal alterations, suggesting a form of “weight-loss memory” within the tissue microenvironment (52). This finding is highly relevant because it indicates that prior obesity may leave durable spatial imprints that continue to influence tumor immune organization and therapeutic response. Human single-cell studies further support the relevance of obesity-associated spatial and cellular heterogeneity. Zhao et al. used single-cell RNA sequencing to reveal a distinct tumor microenvironment landscape in obesity-associated breast cancer, identifying obesity-linked changes in immune and stromal cell populations (160). These data suggest that obesity-associated TIME remodeling is not restricted to animal models but can also be detected in human tumors. Although single-cell RNA sequencing does not always preserve spatial coordinates, when integrated with spatial transcriptomics, multiplex imaging, and spatial metabolomics, it can help define the cellular composition and functional programs underlying obesity-associated metabolic niches. **Table 3** compares the major characteristics of obesity-associated spatial metabolic niches, including their drivers, metabolite profiles, immune composition, functional consequences, and potential therapeutic implications.

**Table 3:** Characteristics and therapeutic implications of spatial metabolic niches in obesity-associated tumors.

Niche type	Major drivers	Metabolite profile	Immune composition	Functional implications	Therapeutic implications
Adipocyte-rich/lipid-enriched niche	Adipocyte proximity, lipid transfer, leptin/adiponectin imbalance, adipose-derived EVs	Fatty acids, cholesterol, lipid droplets, adipokines	Lipid-stressed CD8^+^ T cells, dysfunctional NK cells, lipid-associated TAMs	Supports tumor FAO, nutrient competition, immune-cell metabolic stress	Target lipid uptake, FAO, CD36/FABP pathways, adipokine signaling
Hypoxic/lactate-enriched niche	Vascular dysfunction, rapid tumor expansion, poor perfusion, stromal compression	Lactate, acidic pH, ROS, hypoxia-associated metabolites	Exhausted CD8^+^ T cells, impaired NK cells, Tregs, suppressive macrophages	Suppresses cytotoxicity, promotes immune exclusion, enhances glycolysis	Target lactate transport, MCTs, hypoxia pathways, vascular normalization
TAM/MDSC-dense myeloid niche	Chronic inflammation, microbiota-derived metabolites, chemokines, lipid overload	Arginine depletion, ROS, nitric oxide, inflammatory cytokines, lipid metabolites	TAMs, PMN-MDSCs, M-MDSCs, reduced effector T-cell activity	Drives T-cell suppression, checkpoint dependency, immunotherapy resistance	Target CSF1R, CCR2/CCL2, IL-6/IL-1β, MDSC metabolism, PD-1^+^ TAMs
CAF/ECM-rich stromal barrier niche	TGF-β signaling, CAF activation, ECM deposition, tissue stiffness, adipose remodeling	Collagen-rich ECM, matrix fragments, TGF-β-associated signals	Excluded CD8^+^ T cells, CAFs, TAMs, hypoxia-adapted tumor cells	Restricts immune infiltration, compresses vessels, impairs drug delivery	Target CAF/ECM remodeling, TGF-β signaling, stromal normalization, combination with ICI

CAF, cancer-associated fibroblast; CCL2, C-C motif chemokine ligand 2; CD8+, cluster of differentiation 8-positive; CD36, cluster of differentiation 36; CCR2, C-C chemokine receptor 2; CSF1R, colony-stimulating factor 1 receptor; ECM, extracellular matrix; EVs, extracellular vesicles; FABP, fatty acid-binding protein; FAO, fatty acid oxidation; ICI, immune checkpoint inhibitor; IL-1β, interleukin-1 beta; IL-6, interleukin-6; MCTs, monocarboxylate transporters; MDSCs, myeloid-derived suppressor cells; M-MDSCs, monocytic myeloid-derived suppressor cells; NK cells, natural killer cells; PD-1, programmed cell death protein 1; pH, potential of hydrogen; PMN-MDSCs, polymorphonuclear myeloid-derived suppressor cells; ROS, reactive oxygen species; TAMs, tumor-associated macrophages; TGF-β, transforming growth factor beta; Tregs, regulatory T cells.

Importantly, these obesity-associated niches should be interpreted as dynamic and interconvertible states rather than fixed anatomical compartments. An adipocyte-rich niche may gradually evolve into a hypoxic and lactate-enriched niche when excessive lipid transfer, tumor-cell expansion, and adipose-associated vascular dysfunction increase oxygen demand and impair perfusion. Hypoxia and lactate accumulation can then promote myeloid-cell recruitment, macrophage polarization, MDSC expansion, and immunosuppressive cytokine production, thereby generating TAM/MDSC-dense regions. In parallel, adipocyte-derived factors, tumor-derived extracellular vesicles, TGF-β signaling, inflammatory cytokines, and mechanical stress can activate CAFs and enhance ECM deposition, forming stromal barrier niches that further compress vessels, restrict immune-cell infiltration, and reinforce hypoxia. Thus, vascular dysfunction, metabolic gradients, stromal signaling, adipokine imbalance, and myeloid inflammation may collectively drive niche formation and transition. This dynamic model suggests a feed-forward spatial circuit in which lipid enrichment promotes hypoxia, hypoxia promotes myeloid suppression, and stromal remodeling further stabilizes immune exclusion and therapy resistance.

**Figure 5:**
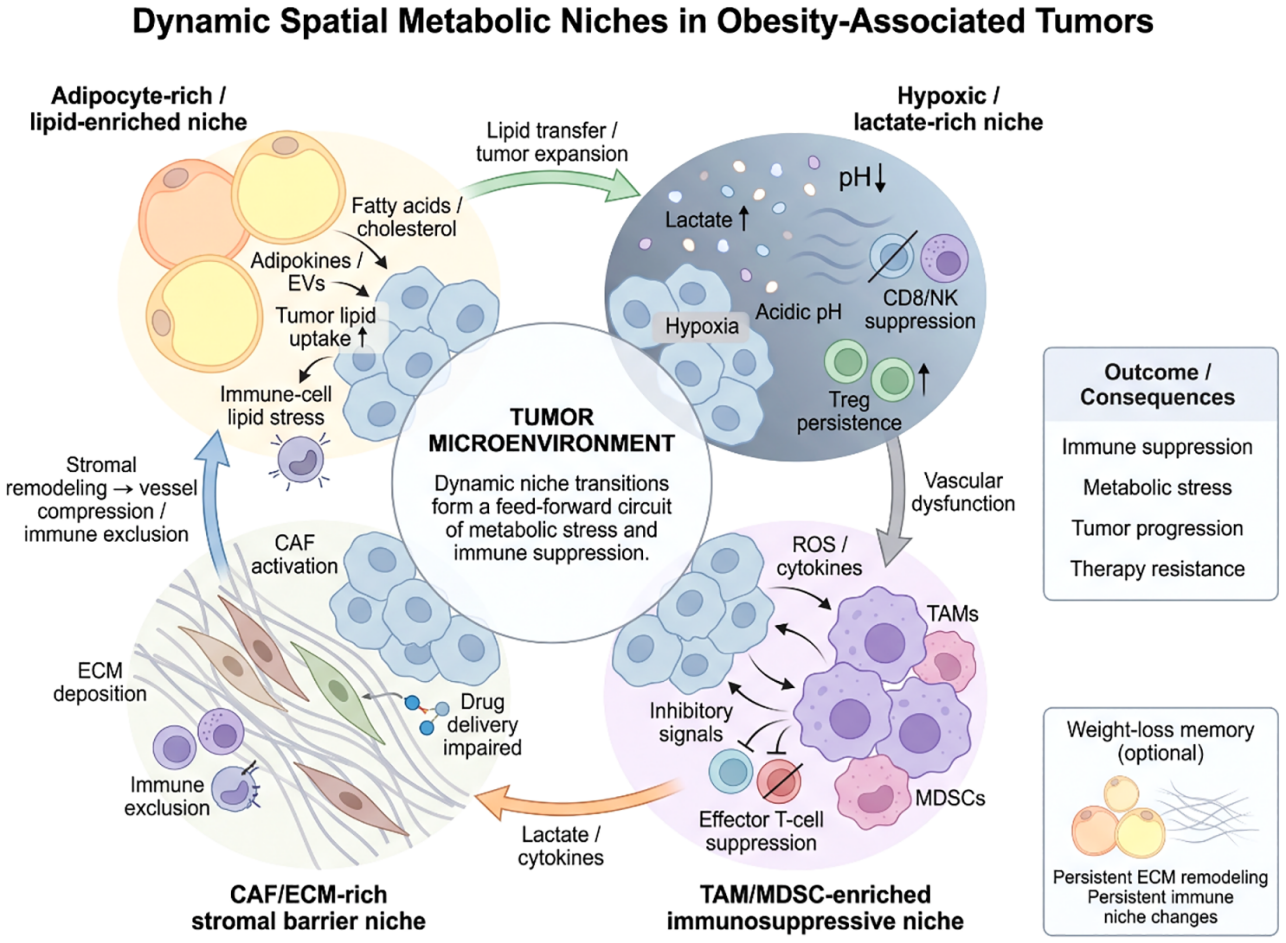
Spatial metabolic niches in obesity-associated tumors. Obesity creates interconnected adipocyte-rich, hypoxic/lactate-enriched, TAM/MDSC-dense, and CAF/ECM-rich niches within tumors. These spatial niches supply lipids and adipokines, suppress CD8+ T-cell and NK-cell function, reinforce myeloid immunosuppression, restrict immune infiltration and drug delivery, and collectively promote tumor progression and therapy resistance. CAF, cancer-associated fibroblast; CD8+, cluster of differentiation 8-positive; ECM, extracellular matrix; EVs, extracellular vesicles; MDSCs, myeloid-derived suppressor cells; NK cells, natural killer cells; pH, potential of hydrogen; ROS, reactive oxygen species; TAMs, tumor-associated macrophages; Treg cells, regulatory T cells.

Collectively, spatial metabolic niches provide a useful framework for understanding how obesity shapes tumor immunity. Adipocyte-rich niches supply lipids and adipokines; hypoxic and lactate-enriched niches suppress cytotoxic immunity; TAM/MDSC-enriched niches mediate myeloid immunosuppression; and CAF/ECM-rich niches restrict immune infiltration and drug delivery. These niches are not isolated compartments but interconnected regions that cooperate to generate a metabolically stressed, immune-suppressive, and therapy-resistant tumor ecosystem. Future studies integrating single-cell sequencing, spatial transcriptomics, multiplex immunofluorescence, spatial proteomics, and imaging mass spectrometry will be essential for mapping these obesity-associated niches and identifying targetable vulnerabilities for immunotherapy. **Figure 5** illustrates how obesity-associated tumor immune remodeling occurs within spatially organized metabolic niches rather than uniformly across the tumor microenvironment. It highlights four interconnected niche types that cooperate to generate a metabolically stressed, immune-suppressive, and therapy-resistant tumor ecosystem.

Although the mechanisms discussed above are presented as a general framework, obesity-associated TIME remodeling is not identical across cancer types. Some features appear broadly shared, including lipid overload, chronic inflammation, myeloid-cell expansion, impaired CD8+ T-cell fitness, adipokine imbalance, and altered response to immune checkpoint blockade (161, 162). However, the dominant immune-metabolic niche may depend strongly on the organ of origin. In breast cancer, obesity-associated mammary adipose tissue can directly shape adipocyte-rich and myeloid-enriched niches, promoting lipid transfer, leptin signaling, TAM/MDSC accumulation, and spatial immune remodeling (19, 128, 148). In colorectal cancer, the gut barrier, mucosal immunity, and microbiota-derived metabolites may be particularly important, linking high-fat diet, dysbiosis, butyrate or amino acid metabolism, and myeloid differentiation (118). In pancreatic cancer, visceral adipose-derived extracellular vesicles and desmoplastic CAF/ECM barriers may interact with poor perfusion to reinforce hypoxia, immune exclusion, and ICB resistance (88). In prostate cancer, obesity may cooperate with androgen-related and MYC-associated metabolic programs to enhance lipid utilization, lactate accumulation, and stromal remodeling (72). By contrast, tumors such as lung cancer may be influenced less by direct adipocyte contact and more by systemic inflammation, myeloid remodeling, CD8+ T-cell infiltration defects, and checkpoint dependency (51). Thus, obesity-associated TIME remodeling should be interpreted as a combination of shared systemic mechanisms and organ-specific immune-metabolic ecology.

## 7 Obesity and immunotherapy response

The relationship between obesity and immunotherapy response is complex and context-dependent. Obesity is generally associated with chronic inflammation, metabolic dysfunction, impaired immune surveillance, and tumor-promoting immunosuppression. However, several preclinical and clinical observations suggest that obese hosts may, in certain settings, display enhanced responses to immune checkpoint inhibitors (ICIs). This apparent contradiction is commonly referred to as the “obesity paradox.”. However, the obesity paradox should be interpreted cautiously because much of the clinical evidence is derived from retrospective analyses that are vulnerable to confounding and selection bias. BMI is an imprecise surrogate for obesity and does not distinguish visceral adiposity, subcutaneous fat, skeletal muscle mass, sarcopenic obesity, cachexia, metabolic health, or systemic inflammation (163–165). In patients with advanced cancer, low BMI may reflect cancer-related weight loss or cachexia rather than a truly metabolically healthy state, which can falsely exaggerate the apparent survival advantage of higher BMI. In addition, differences in treatment selection, immune checkpoint inhibitor exposure, therapeutic dose adjustment, pharmacokinetics, performance status, comorbidities, and corticosteroid or antibiotic use may influence outcomes (166, 167). Therefore, the obesity paradox should not be viewed as a causal protective effect of obesity, but rather as a hypothesis-generating clinical observation that requires prospective validation using body-composition imaging, metabolic biomarkers, inflammatory indices, and immune-spatial profiling. It is also important to distinguish the obesity paradox in immunotherapy response from the relationship between obesity and cancer incidence. At the population level, obesity remains a well-established risk factor for the development and progression of multiple cancers through insulin/IGF-1 signaling, chronic inflammation, adipokine imbalance, altered sex hormone metabolism, and microbiota dysregulation (104, 168). By contrast, the obesity paradox in ICI-treated patients refers to the observation that, among patients who have already developed cancer and received checkpoint blockade, higher BMI or adiposity may sometimes correlate with improved therapeutic outcomes (169, 170). These two concepts should not be conflated: obesity may increase cancer risk and promote baseline immune dysfunction while, in selected treatment contexts, creating checkpoint-dependent immune states that are partially reversible by PD-1/PD-L1 blockade.

Sex hormones and menopausal status may further modify the relationship between obesity and immunotherapy response (171, 172). Obesity alters estrogen, androgen, insulin, leptin, and inflammatory signaling in a sex-dependent manner, and these endocrine changes can influence adipose distribution, immune-cell composition, T-cell exhaustion, macrophage polarization, and tumor immunogenicity (161, 173). In postmenopausal women, adipose tissue becomes an important source of estrogen production, which may be particularly relevant to hormone-sensitive tumors and obesity-associated inflammation. In men, obesity-related changes in androgen–estrogen balance may also affect immune tone and metabolic regulation (3, 165). Therefore, future analyses of the obesity paradox should incorporate sex-stratified and menopausal-status-stratified designs rather than treating obesity as a uniform exposure across all patients.

Importantly, the obesity paradox should not be interpreted as evidence that obesity is biologically beneficial. Rather, it reflects the possibility that obesity creates a distinct immune-metabolic state in which antitumor immunity is suppressed at baseline but may become more dependent on checkpoint-regulated inhibitory pathways, thereby increasing the potential reversibility of immune dysfunction after ICI treatment (174–178).

One important mechanism underlying obesity-associated immunotherapy response involves the PD-1 axis in tumor-associated macrophages. While PD-1 is classically discussed in the context of exhausted T cells, recent evidence indicates that macrophage-expressed PD-1 can also shape antitumor immunity (179, 180). Bader et al. demonstrated that obesity induces PD-1 expression on macrophages, leading to impaired antitumor immune activity and suppression of CD8+ T-cell responses (95). This finding expands the checkpoint paradigm beyond T cells and identifies PD-1-positive TAMs as a myeloid mechanism by which obesity suppresses tumor immunity. It also provides a plausible explanation for why obese tumors may be more responsive to PD-1 blockade in selected contexts: ICI treatment may not only reinvigorate exhausted T cells but also release macrophages from PD-1-mediated functional suppression. Obesity also alters the systemic and local immune landscape during checkpoint blockade. Pingili et al. showed that immune checkpoint blockade reprograms both systemic immune responses and the tumor microenvironment in obesity-associated breast cancer (128). This study highlights that ICI efficacy in obese hosts cannot be understood solely by examining tumor-infiltrating T cells. Instead, therapeutic outcome may depend on coordinated remodeling of peripheral immunity, myeloid populations, cytokine networks, and stromal or metabolic tumor niches. These findings support a broader model in which obesity generates an immune ecosystem that is both dysfunctional and potentially reprogrammable under checkpoint inhibition.

CD8+ T-cell metabolic dysfunction represents another key determinant of ICI response in obesity. Obese tumors can restrict glucose availability, increase lactate accumulation, promote lipid stress, and impair mitochondrial fitness, all of which limit CD8+ T-cell effector function. Such metabolic stress may promote exhaustion-like states characterized by increased expression of inhibitory receptors and reduced cytokine production. In this setting, checkpoint blockade may partially restore T-cell function if sufficient antigen recognition and residual effector capacity remain. However, when metabolic suppression is severe, such as in highly lipid-rich, lactate-enriched, or myeloid-dominated niches, ICI alone may be insufficient. Therefore, obesity can generate both ICI-responsive and ICI-resistant immune states depending on the balance between reversible checkpoint dependency and irreversible metabolic dysfunction.

Cytokines and adipokines further modulate the interaction between obesity and immunotherapy. Obesity-associated elevation of leptin, IL-6, TNF-α, IL-1β, and other inflammatory mediators can reshape T-cell exhaustion, macrophage polarization, MDSC expansion, and stromal remodeling. Dudzinski et al. demonstrated that leptin can augment antitumor immunity in obesity by repolarizing tumor-associated macrophages (127). This study illustrates the dual nature of adipokine signaling: although leptin is often associated with obesity-driven inflammation and tumor progression, it may also influence immune responsiveness by altering macrophage functional states. Thus, adipokine signaling should be interpreted in a context-specific manner rather than as uniformly pro- or antitumor. Obesity-induced tissue remodeling may also influence immunotherapy response before overt tumor development. Lo et al. used single-cell RNA sequencing to show that obesity induces broad transcriptomic alterations in murine mammary glands, affecting epithelial, stromal, and immune compartments (133). These findings suggest that obesity can precondition tissue immune architecture and create a tumor-permissive microenvironment even before malignant transformation. Such pre-existing immune and stromal alterations may later influence whether a tumor develops an inflamed, immune-excluded, or myeloid-dominated phenotype, thereby affecting ICI responsiveness.

**Figure 6:**
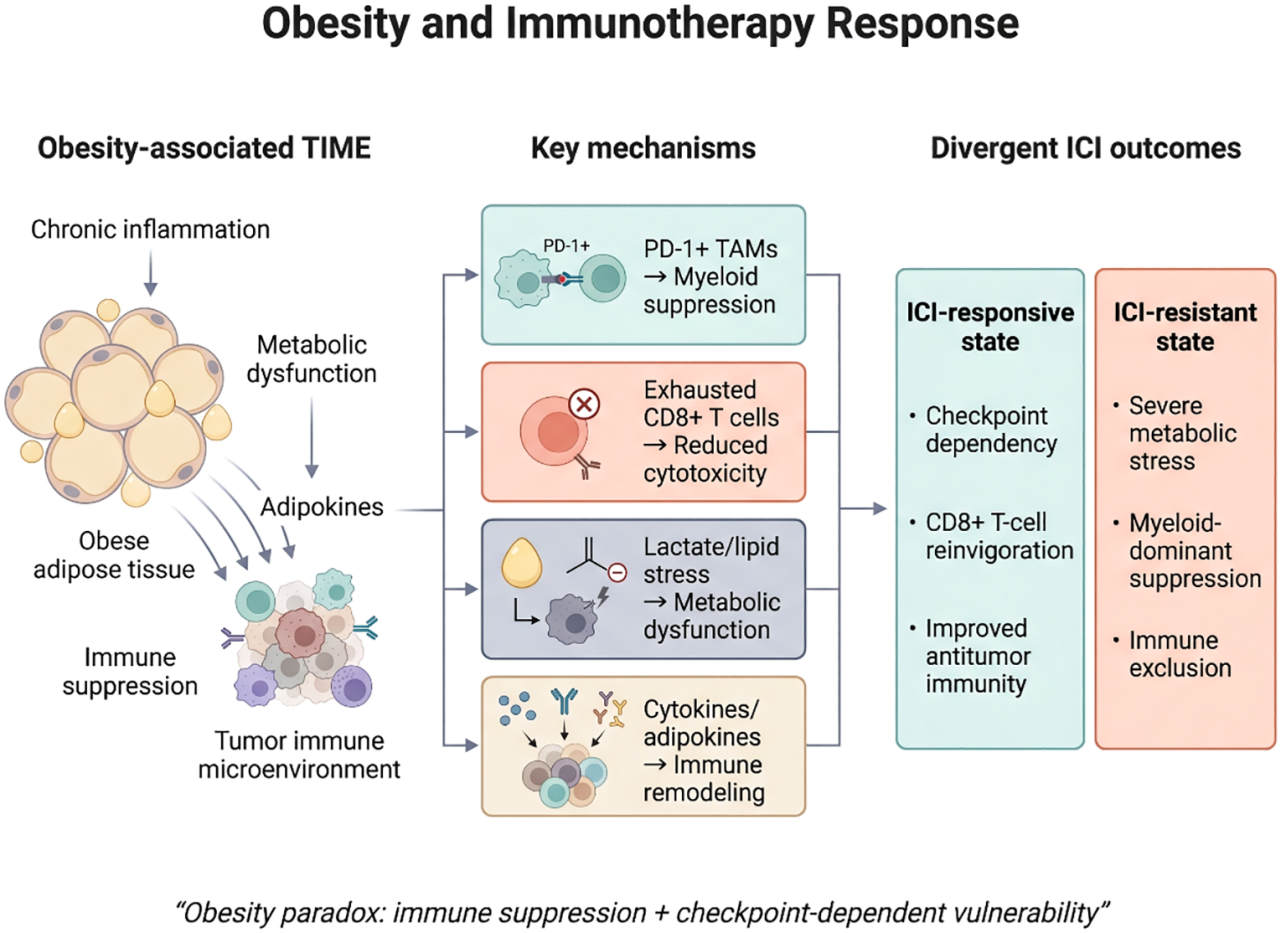
Obesity and immunotherapy response. Obesity creates a metabolically dysfunctional and immunosuppressive TIME characterized by PD-1+ TAMs, exhausted CD8+ T cells, lactate/lipid stress, and cytokine/adipokine remodeling. These changes can produce divergent ICI outcomes, ranging from checkpoint-dependent immune reinvigoration to persistent myeloid suppression, immune exclusion, and therapy resistance. CD8+, cluster of differentiation 8-positive; ICI, immune checkpoint inhibitor; PD-1, programmed cell death protein 1; PD-1+, programmed cell death protein 1-positive; TAMs, tumor-associated macrophages; TIME, tumor immune microenvironment; T cell, T lymphocyte.

Taken together, obesity influences immunotherapy response through multiple interconnected mechanisms, including PD-1-dependent macrophage suppression, CD8+ T-cell exhaustion and metabolic restriction, cytokine and adipokine remodeling, systemic immune reprogramming, and tissue-level immune preconditioning. These mechanisms help explain why obesity may be associated with immune dysfunction and tumor progression while also creating checkpoint-dependent vulnerabilities in selected tumors. Future therapeutic strategies should move beyond BMI alone and incorporate metabolic health, adipose distribution, immune-cell composition, myeloid checkpoint activity, and spatial metabolic niches to identify which obese patients are likely to benefit from ICI monotherapy or require combination approaches targeting metabolism, inflammation, or myeloid suppression. **Figure 6** illustrates the context-dependent relationship between obesity and immunotherapy response. It highlights the obesity paradox, in which obesity suppresses baseline antitumor immunity but may also create checkpoint-dependent vulnerabilities that can be partially reversed by immune checkpoint blockade.

## 8 Therapeutic opportunities

The recognition that obesity reshapes the tumor immune microenvironment through metabolic, inflammatory, stromal, and microbial mechanisms creates new therapeutic opportunities. Instead of viewing obesity only as a clinical risk factor, it may be considered a modifiable biological state that can be targeted to improve antitumor immunity and immunotherapy response (181–184). Therapeutic strategies for obesity-associated tumors may include direct metabolic intervention, combination with immune checkpoint inhibitors (ICIs), lifestyle-based metabolic reconditioning, targeting adipocyte–myeloid crosstalk, and modulation of gut microbiota-derived metabolites.

One important strategy is to combine immunotherapy with metabolic interventions that reverse tumor- or immune-cell metabolic dysfunction. In obese tumors, enhanced fatty acid uptake and fatty acid oxidation (FAO), lactate accumulation, glucose restriction, and mitochondrial stress can suppress cytotoxic T-cell and NK-cell function while supporting tumor cells, tumor-associated macrophages (TAMs), regulatory T cells, and myeloid-derived suppressor cells (MDSCs) (81, 185–188). Therefore, targeting FAO, lipid uptake, lactate production, or monocarboxylate transporter (MCT)-mediated lactate transport may help normalize the metabolic landscape of the obese tumor immune microenvironment. Such approaches may be particularly valuable when combined with ICIs, because checkpoint blockade requires metabolically competent effector immune cells to generate durable antitumor responses. In this framework, metabolic therapy would not simply inhibit tumor growth directly, but would also convert metabolically hostile immune niches into more permissive environments for T-cell reinvigoration.

Weight-loss, dietary modification, and exercise represent another set of clinically relevant interventions. These approaches may reduce systemic inflammation, improve insulin sensitivity, normalize adipokine profiles, decrease ectopic lipid accumulation, and restore immune-cell metabolic fitness. Piening et al. showed that obesity-related T-cell dysfunction impairs immunosurveillance and increases cancer risk, and their findings further suggest that dietary or weight-loss interventions can influence obesity-associated immune dysfunction (189). These results support the concept that metabolic health, rather than body mass index alone, may determine antitumor immune competence. However, the timing, magnitude, and type of intervention are likely critical. For example, weight loss before tumor development may differ biologically from weight loss during advanced cancer or immunotherapy. Moreover, rapid weight reduction, malnutrition, or sarcopenia could negatively affect treatment tolerance. Therefore, future clinical studies should define how intentional weight management, exercise programs, and metabolic optimization can be safely integrated with cancer immunotherapy.

Targeting the adipocyte–TAM–MDSC axis is another promising direction. Obesity-associated adipose tissue releases fatty acids, leptin, inflammatory cytokines, and extracellular vesicles that promote myeloid recruitment and suppressive polarization. TAMs and MDSCs can then inhibit CD8+ T-cell infiltration, induce T-cell apoptosis, secrete immunosuppressive mediators, and reinforce stromal barriers. Therapeutic strategies may include blocking CCL2/CCR2-mediated myeloid recruitment, inhibiting CSF1R-dependent macrophage survival, targeting MDSC-associated lipid metabolism, reducing IL-1β or IL-6 signaling, or reprogramming TAMs toward immune-stimulatory states (190). Li F et al. reported that EGCG alleviates obesity-exacerbated lung cancer progression through the STAT1/SLC7A11 pathway and gut microbiota modulation (51). This study suggests that interventions capable of simultaneously regulating tumor-intrinsic stress pathways, inflammatory signaling, and microbial composition may be particularly useful in obesity-associated cancer.

The gut microbiota provides an additional therapeutic target linking diet, systemic metabolism, and tumor immunity. High-fat diet-induced dysbiosis can alter microbial metabolites, including short-chain fatty acids, bile acids, amino acids, and other immunomodulatory molecules. These metabolites can influence myeloid differentiation, T-cell function, epithelial barrier integrity, and systemic inflammation. Shao et al. demonstrated that a high-fat diet promotes colitis-associated tumorigenesis by altering gut microbial butyrate metabolism (49). This work highlights the importance of microbial metabolites as mediators of obesity-associated tumor-promoting inflammation. Therapeutic approaches such as dietary fiber supplementation, prebiotics, probiotics, postbiotics, fecal microbiota transplantation, or targeted metabolite modulation may help restore a more favorable immune-metabolic state. However, microbiota-based interventions should be developed carefully because the effects of specific microbial metabolites may vary according to tumor type, anatomical site, host diet, and immune context. Several combination strategies may be particularly attractive. First, ICI plus metabolic normalization could be used to restore CD8+ T-cell metabolic fitness in lipid- or lactate-rich tumors. Second, ICI plus myeloid-targeted therapy may overcome obesity-associated TAM/MDSC-mediated immune suppression. Third, dietary or microbiota-based interventions may be used to reduce systemic inflammation and improve immune responsiveness before or during immunotherapy. Fourth, targeting tumor-intrinsic antioxidant or stress-response pathways, such as the STAT1/SLC7A11 axis, may increase tumor vulnerability while reducing obesity-driven immune evasion. These strategies should be guided by biomarkers, including visceral adiposity, insulin resistance, circulating inflammatory cytokines, leptin/adiponectin ratio, gut microbial metabolites, TAM/MDSC abundance, CD8+ T-cell metabolic status, and spatial metabolic niche signatures.

Overall, therapeutic opportunities in obesity-associated tumors should move beyond generalized weight reduction and toward precision metabolic immuno-oncology. The goal is not merely to reduce body weight, but to reprogram the systemic and local immune-metabolic environment that supports tumor progression and immunotherapy resistance. By integrating lifestyle intervention, metabolic targeting, myeloid reprogramming, microbiota modulation, and ICIs, future treatment strategies may convert obesity-associated immune suppression into a therapeutically targetable vulnerability. Because obesity-associated immune suppression is driven by interconnected metabolic, inflammatory, microbial, and stromal mechanisms, therapeutic strategies may require combination approaches rather than single-pathway targeting. **Table 4** summarizes potential interventions aimed at restoring antitumor immunity and improving immunotherapy efficacy in obesity-associated cancers.

**Table 4:** Therapeutic strategies targeting obesity-associated TIME.

Strategy	Target/mechanism	Potential benefit	References (PMID)
Metabolic + ICI combination	FAO inhibition, lactate/MCT targeting	Restore CD8+ T-cell function & improve ICI efficacy	Ringel 2020 (33301708); Boufaied 2024 (38831751)
Weight-loss/Diet/Exercise	Systemic metabolic reprogramming	Reduce inflammation, improve immune fitness	Piening 2024 (38565540)
Adipocyte–TAM–MDSC axis targeting	CCL2/CCR2, CSF1R, MDSC FAO, leptin signaling	Reduce immune suppression, enhance antitumor immunity	Li F 2023 (37451475); Xue 2025 (41338178)
Microbiota modulation	SCFA, bile acids, amino acids	Reprogram myeloid differentiation & T-cell activity	Shao 2023 (37781523)
Tumor-intrinsic stress pathways	STAT1/SLC7A11, oxidative stress	Sensitize tumors to ICI & metabolic stress	Li F 2023 (37451475)

CCL2, C-C motif chemokine ligand 2; CD8+, cluster of differentiation 8-positive; CCR2, C-C chemokine receptor 2; CSF1R, colony-stimulating factor 1 receptor; FAO, fatty acid oxidation; ICI, immune checkpoint inhibitor; MCT, monocarboxylate transporter; MDSC, myeloid-derived suppressor cell; PMID, PubMed identifier; SCFA, short-chain fatty acid; SLC7A11, solute carrier family 7 member 11; STAT1, signal transducer and activator of transcription 1; TAM, tumor-associated macrophage; TIME, tumor immune microenvironment.

## 9 Biomarkers and clinical stratification

Effective clinical management of obesity-associated tumors requires robust biomarkers to stratify patients based not only on body mass index (BMI) but also on the underlying metabolic, immunological, and spatial characteristics of the tumor immune microenvironment (TIME) (191–194). Traditional anthropometric measures such as BMI provide a crude estimate of adiposity but fail to capture the complexity of metabolic health, visceral fat distribution, and systemic inflammatory status. Therefore, integrated biomarker strategies are essential to predict tumor progression, immune dysfunction, and response to immunotherapy in obese patients. Metabolic and inflammatory indicators represent a key dimension for patient stratification. These include circulating cytokines and adipokines such as IL-6, TNF-α, IL-1β, leptin, and adiponectin, as well as insulin resistance markers, lipid profiles, and systemic metabolite concentrations. Ringel et al. demonstrated that metabolic competition between tumor cells and CD8+ T cells serves as a mechanistic predictor of antitumor immune fitness (16). These findings suggest that metabolic markers—particularly those reflecting nutrient availability, fatty acid oxidation, and glycolytic restriction—can be used to assess immune competence within the tumor microenvironment.

Immune-metabolic signatures provide an additional layer of granularity. Quantifying the abundance, functional state, and metabolic activity of key immune populations—including CD8+ effector T cells, regulatory T cells (Tregs), tumor-associated macrophages (TAMs), and myeloid-derived suppressor cells (MDSCs)—can inform on the immunosuppressive or immunotherapy-responsive potential of the tumor. Integrating these cellular metrics with metabolic profiles enables the identification of patients whose antitumor immunity may be impaired by obesity-induced metabolic competition or who may benefit from metabolic or checkpoint-targeted interventions. Spatial profiling of metabolites and immune cells further enhances predictive accuracy. Zhao et al. employed single-cell and spatial transcriptomics to reveal spatially resolved immune-metabolic biomarkers in human obesity-associated breast tumors (160). Their study highlighted that distinct tumor regions—such as lipid-rich, hypoxic, or myeloid-dense niches—display unique patterns of immune suppression and metabolic adaptation. Spatially resolved biomarkers can therefore provide actionable insights into the heterogeneity of obesity-associated TIME, enabling clinicians to identify metabolically vulnerable niches and guide local or systemic therapeutic interventions.

The combination of systemic, cellular, and spatial biomarkers supports a precision medicine framework for obese cancer patients. A multi-dimensional stratification strategy might include: (1) anthropometric and visceral fat measures, (2) metabolic health indicators such as insulin sensitivity, lipid profiles, and circulating metabolites, (3) immune cell composition and functional metabolic signatures, and (4) spatially resolved metabolite and immune-cell distributions within the tumor. By integrating these biomarkers, clinicians can better predict which patients are likely to respond to immune checkpoint blockade, metabolic interventions, or combination therapies, and tailor treatment plans to mitigate obesity-associated immune dysfunction.

In summary, biomarker-driven clinical stratification in obesity-associated tumors requires an integrated approach that considers systemic metabolic health, immune-cell metabolic and functional states, and the spatial organization of tumor-immune-metabolic niches. Such strategies promise to improve the selection of patients for immunotherapy, identify targets for metabolic or myeloid-directed therapies, and ultimately enhance therapeutic efficacy in the context of obesity-induced tumor immune remodeling.

## 10 Challenges and future perspectives

Despite rapid progress in understanding the relationship between obesity, tumor metabolism, and antitumor immunity, several major challenges remain. First, body weight alone is an insufficient indicator of obesity-associated immune-metabolic risk. Most clinical studies use body mass index (BMI) as the primary measure of obesity, but BMI does not distinguish between visceral adiposity, subcutaneous fat, muscle mass, metabolic health, insulin resistance, systemic inflammation, or adipose tissue dysfunction. Two patients with the same BMI may have markedly different immune and metabolic phenotypes. Therefore, future studies should move beyond BMI and incorporate more informative measures, including visceral fat area, waist-to-hip ratio, insulin sensitivity, lipid profiles, circulating cytokines, adipokine ratios, sarcopenia, and metabolic syndrome status. This distinction is particularly important for interpreting the obesity paradox in immunotherapy, because improved ICI response may reflect specific immune-metabolic states rather than obesity itself.

Second, animal models do not fully capture the heterogeneity of human obesity and cancer. High-fat diet models are widely used to study obesity-associated tumor immunity, but they vary substantially in diet composition, feeding duration, mouse strain, sex, microbiota background, tumor implantation method, and timing of obesity induction. These variables can strongly influence tumor growth, immune infiltration, myeloid-cell expansion, and response to immune checkpoint blockade. Moreover, diet-induced obesity in mice may not reproduce the complex clinical features of human obesity, such as long-term metabolic syndrome, medication use, aging, sex hormone changes, microbiome diversity, or comorbidities. Pingili et al. showed that immune checkpoint blockade can reprogram both systemic immune responses and the tumor microenvironment in obesity-associated breast cancer, highlighting the importance of analyzing systemic and local immunity together (128). Future experimental designs should better model clinically relevant obesity phenotypes and include both male and female animals, multiple diet formulations, genetically and diet-induced obesity models, and validation in human tumor cohorts.

Third, the causal relationships among spatial metabolic niches, immune suppression, and therapy response remain incompletely defined. Single-cell sequencing, spatial transcriptomics, multiplex imaging, and spatial metabolomics have revealed that obesity-associated tumors contain lipid-rich, hypoxic, myeloid-dense, and stromal-barrier regions. However, spatial association does not necessarily prove causality. It remains unclear whether specific niches directly cause immune exclusion or whether they emerge as a consequence of tumor progression, inflammation, or therapy-induced selection. Kuziel et al. demonstrated that formerly obese mice retain persistent mammary gland and tumor microenvironment alterations, including stromal and extracellular matrix remodeling, even after weight normalization (52). This finding suggests that obesity may leave durable tissue-level “memory” that continues to influence tumor immunity. Future studies should combine spatial mapping with functional perturbation, lineage tracing, metabolic flux analysis, and longitudinal sampling to determine which spatial niches are causally responsible for immune dysfunction and immunotherapy resistance.

Fourth, obesity-associated tumor immunity is highly dependent on tumor type, anatomical site, sex, diet composition, and gut microbiota. Tumors arising in adipose-rich organs, such as breast, pancreas, colon, ovary, and prostate, may be more directly influenced by local adipocyte–tumor interactions than tumors in less adipose-associated tissues. Sex differences may also shape obesity-associated cancer immunity through differences in fat distribution, sex hormones, immune tone, and metabolic regulation. Diet composition is another key variable: saturated fats, unsaturated fats, carbohydrates, fiber, and micronutrients may have distinct effects on gut microbiota, systemic inflammation, and antitumor immunity even when they produce similar weight gain. Gut microbiota further complicates this relationship by converting dietary components into metabolites that regulate myeloid differentiation, T-cell function, epithelial barrier integrity, and response to immunotherapy. Therefore, future studies should avoid treating obesity as a uniform exposure and instead define specific obesity subtypes based on diet, metabolic health, microbiota, and tissue context.

Fifth, the clinical translation of obesity-targeted immunometabolic strategies requires better integration of multi-omics data. Current evidence suggests that obesity can regulate tumor metabolism, immune-cell function, adipocyte-derived signaling, stromal remodeling, and microbiota-derived metabolites. However, these layers are often studied separately. A more comprehensive translational framework should integrate bulk transcriptomics, single-cell RNA sequencing, spatial transcriptomics, proteomics, metabolomics, lipidomics, microbiome profiling, imaging-based adiposity assessment, and clinical immunotherapy outcomes. Xue et al. showed that extracellular vesicles from obese visceral adipose tissue promote pancreatic cancer progression and resistance to immune checkpoint blockade, illustrating how systemic adipose-derived signals can influence distant tumors and therapeutic response (88). Such findings highlight the need for cross-tissue and cross-scale analyses that connect adipose tissue, gut microbiota, circulating metabolites, tumor-intrinsic programs, and immune-cell states.

Finally, future therapeutic development should focus on precision metabolic immuno-oncology rather than generalized weight reduction alone. Weight loss, dietary modification, exercise, metabolic drugs, microbiota modulation, myeloid-targeted therapy, and immune checkpoint blockade may each influence obesity-associated tumor immunity, but their optimal timing and combination remain unclear. For example, metabolic intervention before tumor development may prevent immune dysfunction, whereas intervention during advanced cancer therapy may need to restore effector T-cell function without inducing malnutrition or sarcopenia. Similarly, targeting adipose-derived extracellular vesicles, TAM/MDSC metabolism, lactate transport, fatty acid oxidation, or inflammatory cytokines may enhance ICI efficacy in selected obese patients. Future clinical trials should stratify patients by metabolic health, visceral adiposity, immune-cell composition, spatial metabolic niches, and microbiota features to identify those most likely to benefit from combination approaches.

In summary, the field must address several key challenges: distinguishing body weight from metabolic health, improving animal-to-human translation, establishing causal links between spatial niches and immune dysfunction, accounting for tumor- and host-specific heterogeneity, and integrating multi-omics data into clinically actionable biomarkers. Addressing these issues will help transform obesity from a broad epidemiological risk factor into a mechanistically defined and therapeutically targetable modifier of tumor immunity and immunotherapy response.

## 11 Conclusion

Obesity is increasingly recognized as more than an epidemiological risk factor for cancer. It represents a systemic metabolic and inflammatory state that actively reshapes the tumor immune microenvironment. Through insulin resistance, lipid overload, adipokine imbalance, chronic inflammation, gut microbiota dysregulation, and adipose-derived signaling, obesity reprograms both tumor cells and immune cells. These systemic alterations are translated into local tumor ecosystems characterized by enhanced nutrient competition, lipid accumulation, lactate enrichment, myeloid expansion, stromal remodeling, and impaired cytotoxic immune function.

A central feature of obesity-associated tumor immunity is the formation of spatially organized immunosuppressive metabolic niches. Adipocyte-rich regions provide fatty acids, adipokines, and extracellular vesicles that support tumor metabolic adaptation. Hypoxic and lactate-enriched regions weaken CD8+ T-cell and NK-cell activity. TAM- and MDSC-dense regions reinforce myeloid-mediated immune suppression, whereas CAF- and ECM-rich stromal barriers restrict immune-cell infiltration and drug delivery. These niches interact with one another to create a tumor microenvironment that is metabolically favorable for cancer cells but functionally hostile to antitumor immunity. Obesity also alters the metabolic and functional states of multiple immune-cell populations. CD8+ T cells may undergo glycolytic restriction, lipid stress, mitochondrial dysfunction, and exhaustion-like changes. Tregs may exploit lipid-rich environments to sustain immunosuppressive activity. TAMs can acquire lipid-associated, M2-like, or PD-1-dependent suppressive phenotypes, while MDSCs expand under obesity-associated inflammatory and microbiota-derived signals. Together, these changes suppress baseline antitumor immunity and promote tumor progression.

At the same time, the relationship between obesity and immunotherapy response is context-dependent. The so-called obesity paradox reflects the possibility that obesity-induced immune dysfunction may increase reliance on checkpoint pathways such as PD-1/PD-L1 in certain tumors, thereby creating a state that is partially reversible by immune checkpoint blockade. However, this effect is not universal and depends on tumor type, sex, adipose distribution, metabolic health, myeloid composition, gut microbiota, and the spatial organization of immune-metabolic niches. Therefore, obesity should not be considered a simple positive or negative predictor of immunotherapy response, but rather a complex modifier of tumor immunity. Future therapeutic strategies should aim to convert obesity-associated immune-metabolic dysfunction into a targetable vulnerability. Combining immune checkpoint inhibitors with metabolic interventions, myeloid reprogramming, microbiota modulation, lifestyle optimization, or adipose-derived signaling blockade may improve therapeutic outcomes in selected patients. Importantly, clinical stratification should move beyond BMI and incorporate visceral adiposity, metabolic health, cytokine and adipokine profiles, immune-cell metabolic signatures, and spatial biomarkers. By integrating systemic metabolism with local tumor immune ecology, future studies may enable more precise and effective immunotherapy strategies for patients with obesity-associated cancers.
